# Interface Diffusion and Compatibility of (Ba,La)FeO_3−δ_ Perovskite Electrodes in Contact with Barium Zirconate and Ceria

**DOI:** 10.1021/acsami.3c13013

**Published:** 2023-10-20

**Authors:** Alessandro Chiara, Giulia Raimondi, Rotraut Merkle, Joachim Maier, Claudio Ventura Bordenca, Candida Pipitone, Alessandro Longo, Francesco Giannici

**Affiliations:** † Dipartimento di Fisica e Chimica, Università di Palermo, 90128 Palermo, Italy; ‡ Max Planck Institute for Solid State Research, 70569 Stuttgart, Germany; § Istituto per lo Studio dei Materiali Nanostrutturati (ISMN)-CNR, UOS Palermo, 90146 Palermo, Italy; ∥ European Synchrotron Radiation Facility, 38000 Grenoble, France

**Keywords:** solid oxide fuel cells, electrolyzers, perovskites, cathodes, X-ray microscopy, XRD, XANES

## Abstract

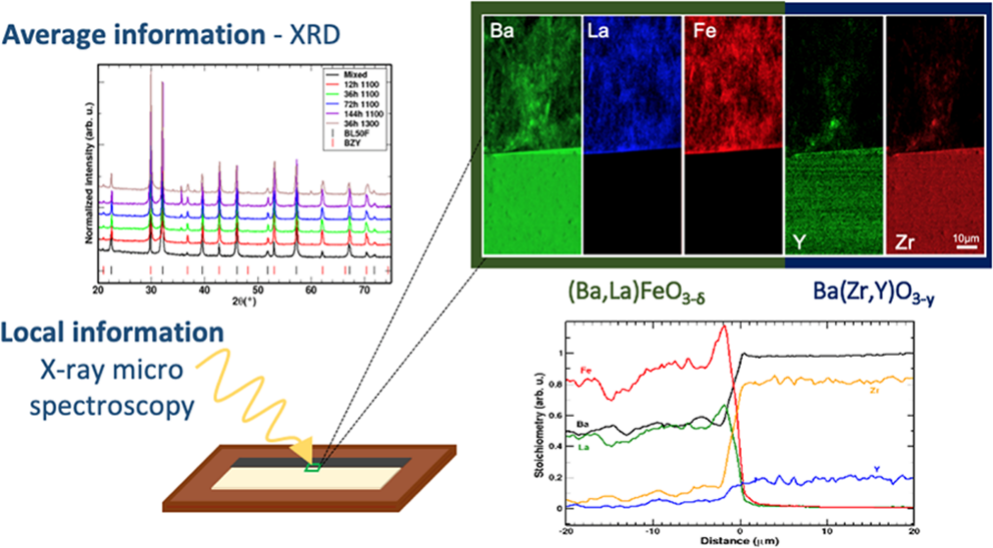

Ba_1–*x*_La_*x*_FeO_3−δ_ perovskites (BLF) capable of conducting electrons, protons, and oxygen ions are promising oxygen electrodes for efficient solid oxide cells (fuel cells or electrolyzers), an integral part of prospected large-scale power-to-gas energy storage systems. We investigated the compatibility of BLF with lanthanum content between 5 and 50%, in contact with oxide-ion-conducting Ce_0.8_Gd_0.2_O_2−δ_ and proton-conducting BaZr_0.825_Y_0.175_O_3−δ_ electrolytes, annealing the electrode–electrolyte bilayers at high temperature to simulate thermal stresses of fabrication and prolonged operation. By employing both bulk X-ray diffraction and synchrotron X-ray microspectroscopy, we present a space-resolved picture of the interaction between electrode and electrolyte as what concerns cation interdiffusion, exsolution, and phase stability. We found that the phase stability of BLF in contact with other phases is correlated with the Goldschmidt tolerance factor, in turn determined by the La/Ba ratio, and appropriate doping strategies with oversized cations (Zn^2+^, Y^3+^) could improve structural stability. While extensive reactivity and/or interdiffusion was often observed, we put forward that most products of interfacial reactions, including proton-conducting Ba(Ce,Gd)O_3−δ_ and mixed-conducting (Ba,La)(Fe,Zr,Y)O_3−δ_, may not be very detrimental for practical cell operation.

## 1 Introduction

The necessity of a defossilized energy sector and the population’s awareness toward the environmental crisis are growing. This requires developing a future energy landscape grid using renewable and clean energy carriers like hydrogen. The latter finds application in fuel cells (FCs), which interconvert electrical energy and chemical energy with high efficiency. Among the different types of fuel cell technologies available nowadays, the one exploiting protonic conduction, the proton ceramic fuel cell (PCFC), is emerging as a potential next-generation electrochemical converter and storage device.^1−9^ PCFCs, compared to solid oxide fuel cells (SOFC), have (i) the advantage to work at a lower temperature (400–600 °C) thanks to the lower activation energy for proton transport compared to oxide-ion transport, which will decrease degradation processes and (ii) water is produced at the cathode side; therefore, the H_2_ is not diluted allowing for high fuel utilization, and direct production of dry hydrogen when operated in electrolysis mode.

PCFCs are based on proton-conducting ceramic electrolytes, most frequently acceptor-doped barium zirconates and cerates.^6,8,10−12^ The cathode materials ideally combine electronic and protonic conductivity and belong to the group of simple perovskites ABO_3_, higher-order ones (double perovskites, Ruddlesden–Popper, etc.), brownmillerites A_2_B_2_O_5_,^13,14^ and references therein. For the sake of simplicity, in the following, we label such mixed-conducting oxides as cathodes, although such an electrode would act as an anode when the cell operates in the electrolysis mode.

One of the key points for fabricating a performant SOFC or PCFC is the compatibility between cathode and electrolyte.^10,15^ The relatively high operational temperature often leads to degradation of the materials involved, such as cation demixing, atomic species segregation, and secondary phases.^16−21^ These processes can strongly influence cell performance, since insulating phases can form, decreasing the ionic and electronic conductivity across the cell. When cation interdiffusion leads to the formation of new phases (the classical example is La_2_Zr_2_O_7_ in SOFC at the contact between LSM and YSZ),^22^ this is often detrimental as the new structure typically has inferior transport properties, and may also cause problems arising from volume changes or differences in thermal expansion. Such situations need to be recognized and avoided by tuning the material compositions and/or applying buffer layers.

The investigation of material compatibility and the respective aging and degradation processes of ceramic devices operated for a very long time at elevated temperatures is challenging for several reasons. On the one hand, processes occurring over many thousand hours at operating temperatures need to be mimicked by experiments running over tens or hundreds of hours at higher temperatures. On the other hand, the different techniques used for investigation are often complementary (e.g., averaging over large sample volume in X-ray diffraction (XRD) vs space-resolved micro-XAS) and also imply some differences in sample geometry and preparation. This can make it difficult to derive a consistent overall picture.

We first applied X-ray microspectroscopy to study the chemical and structural compatibility between various oxide materials for electrolytes (either proton- or oxygen-conducting) and oxygen electrodes such as (La,Sr)(Fe,Co,Cu)O_3−δ_.^19,20,23−25^ Recent applications of more conventional techniques (both X-ray diffraction and electron microscopy) were reviewed.^26^ X-ray fluorescence (XRF) with a hard X-ray synchrotron beam has also been used recently to follow interdiffusion at the micrometer scale across a GDC interlayer between the La_0.6_Sr_0.4_Co_0.2_Fe_0.8_O_3_ oxygen electrode and YSZ electrolyte.^27^

For SOFC, it has been shown that also an applied DC bias (simulating actual fuel cell operation) can affect the processes at the interface.^28^ However, the specific mechanisms are rather complex, often starting with segregation of La or Sr, which may then trigger further reactions with the fluorite (Zr,Ce)O_2_ electrolyte.^29−31^ This situation may also differ from the phenomena in PCFCs, where mainly interfacial reactions between two perovskite phases take place. Therefore, in this investigation, we focus on interfacial compatibility in the absence of DC bias.

Here, we focus on the compatibility with Ba-rich cathode materials since they are most frequently employed in PCFCs.^13,14^ Barium ferrites were chosen as prototypical cathode materials: Ba_0.95_La_0.05_FeO_3-δ_ (BL5F—a minimum La dopant content of 0.05 is necessary to prevent its transformation to the hexagonal perovskite structure^32^), Ba_0.75_La_0.15_FeO_3−δ_ (BL15F), and Ba_0.5_La_0.5_FeO_3−δ_ (BL50F) were placed in contact with electrolytes for SOFC or PCFC (Ce_0.8_Gd_0.2_O_2−δ_ (GDC) and BaZr_0.825_Y_0.175_O_3−δ_ (BZY)). The structure and electronic properties of these barium ferrites have recently been investigated and correlated with proton uptake.^33,34^ GDC is both an oxide-ion conductor for intermediate temperature SOFC, and it has been found recently to decrease degradation also when used as an interlayer in PCFC.^35^ While the details of its beneficial effect are still under investigation, it is important to note that the GDC interlayer in PCFC in ref (35) is not continuous and thus still allows for proton transfer from the electrolyte to the cathode (despite its very low bulk proton conductivity, but porous ceria also develops surface protonic transport^36^). The other electrolyte that we apply, BZY, is the parent electrolyte material for PCFC.^11,12^ While this electrolyte is a Ba-rich perovskite similar to the Ba_1–*x*_La_*x*_FeO_3−δ_ cathode materials, this does not automatically imply the absence of interfacial reactions. A detailed investigation with high spatial resolution and sensitivity including additional structural/chemical information from the X-ray absorption edge structure is necessary since Ba-rich perovskites in particular are often close to their stability limits with respect to composition.

## 2 Experimental Section

### 2.1 Powder Synthesis

Ba_0.95_La_0.05_FeO_3_ (BL5F), Ba_0.85_La_0.15_FeO_3_ (BL15F), and Ba_0.50_La_0.50_FeO_3_ (BL50F) were prepared from aqueous nitrate solutions^37^ and calcined at 1000 °C for 8 h. Ba_1.015_Zr_0.825_Y_0.175_O_3_ (BZY) was prepared by a solid-state reaction: stoichiometric amounts of BaZrO_3_ (Alfa Aesar), Zr_0.90_Y_0.10_O_2_ (TZ10, Tosoh), and Y_2_O_3_ (Alfa Aesar) were mixed. Then, 0.5 wt % of NiO (Alfa Aesar, 99%) was added as a sintering aid. The mixed powders were first wet-milled in *n*-propanol for 24 h in a planetary mill (Fritsch Pulverisette 5) and then calcined in air at 1500 °C for 4 h. Ce_0.8_Gd_0.2_O_2_ (GDC) powder (fuel cell materials) was used as received. GDC and BZY dense pellets (ρ_rel_ ≥ 95%) were prepared by isostatic pressing at 4.1 kbar for 5 min and sintered at 1500 °C for 10 and 5 h, respectively.

### 2.2 X-ray Diffraction (XRD)

XRD patterns were acquired with a Panalytical Empyrean diffractometer in Bragg–Brentano geometry using Ni-filtered Cu Kα radiation. No evidence of secondary phases is detected in the XRD patterns of the as-synthesized materials. XRD was then used to evaluate the reactivity of mixed cathode/electrolyte powders. The samples were prepared by mixing in an agate mortar the cathode and electrolyte powders (1:1 volume ratio) and a few drops of isopropanol, which is then evaporated at 60 °C. The BZY powders are obtained by crushing and ball-milling a previously sintered pellet (1500 °C for 4 h, which are required for proper incorporation of the Y dopant into BZ) in order to be consistent with the preparation of the X-ray microscopy samples presented below. For the X-ray diffraction experiment, pellets with a diameter of 1 cm were prepared for each couple by isostatically pressing ca. 250 mg of mixed powders at 4.1 kbar and then annealing at 1100 °C for either 12, 36, 72, or 144 h; one further pellet was annealed at 1300 °C for 36 h. If not specified otherwise, reported data refer to treatments at 1100 °C. After annealing, the pellets were polished. Rietveld refinement was carried out with GSAS-II:^38^ the achieved goodness of fit (*R*_wp_) was around 5% for all samples. The uncertainty on all reported lattice parameters is on the order of 0.001 Å for major phases and 0.004 Å for minor phases. Rietveld refinements are reported in the Supporting Information (SI).

### 2.3 X-ray Microspectroscopy

The cathode/electrolyte couples were prepared by surrounding the GDC or BZY dense electrolyte pellet with the cathode powder and then isostatic pressing (4.1 kbar for 5 min) to create a core/shell structure.

The core–shell pellets were annealed at 1100 °C for 72 h. The annealed electrolyte–cathode couples were then embedded in epoxy resin and cut into slices to expose the cross section. Scanning electron microscope (SEM) images are shown in the SI.

X-ray absorption spectroscopy (XAS) using a synchrotron microbeam was carried out at beamline ID21 of the European Synchrotron Radiation Facility, Grenoble, France,^39^ at the Fe K-edge (7.1 keV) and Gd L_3_-edge (7.2 keV). The beam size was 800 nm × 330 nm (H × V), with a flux of 3 × 10^10^ ph/s. Data reduction and concentration maps were carried out with PyMca 5.6.7.^40^ Further details on measurement geometry, resolution, and data analysis are found in refs (20,23−25).^20,23−25^

## 3 Results and Discussion

### 3.1 General Remarks

Before we move into the detailed discussion of the experimental results, the phenomena that need to be considered for interfaces between different oxides are briefly summarized here. In general, diffusion between different phases is driven by both entropic and enthalpic effects. The configurational entropy from intermixing cations (and other point defects such as oxygen vacancies) between different phases is unavoidably present as a driving force. Its thermodynamic effect increases with higher temperature; this needs to be considered when extrapolating from high-T short-term experiments to lower-T long-term annealing. As long as no new phases are formed, the effects of such cation interdiffusion on transport properties are often not very detrimental, e.g., an increase of electronic conductivity in the electrolyte limited to the proximity to the cathode may even extend the reactive zone for the oxygen reduction reaction. There may also be an enthalpic driving force for such an interdiffusion, although less generalizable and stemming from chemical effects (e.g., decrease of ion size mismatch, or acid–base-type interactions between transferred cation and matrix oxide). We will make use of these concepts in the discussion of the present results. Such aspects have been recognized to play a role in the dopant distribution in the BaFeO_3_–BaCeO_3_ system.^41^

In addition to the driving forces, the amount of interdiffusion is determined by the mobilities of the cations in and between the respective oxide phases. In the materials considered here (oxides with perovskite and fluorite structure), cation diffusion is typically slow, with bulk diffusion being orders of magnitude lower than diffusion along grain boundaries and interfaces, as shown in Figure S1 (all diffusion coefficients are self/tracer diffusion coefficients; if suitable isotopes are unavailable, chemically similar “impurity cations” were used). Interestingly, the diffusivities of A-site and B-site cations in perovskites are often rather similar, indicating a diffusion mechanism via a common point defect such as A-site vacancies.^42−44^ Taking the averaged range of bulk diffusivities of (Ba,Sr,La)(Fe,Co)O_3–*x*_ perovskites (marked by blue bar in Figure S1), a cation diffusion length of 0.3–3 μm is expected after 12 h at 1100 °C. The grain boundary diffusivities, on the other hand, result in significantly longer diffusion lengths. In the polycrystalline samples under investigation, the results discussed in the following suggest that a mixed bulk/GB control regime is effective. In this respect, XRD is sensitive to large composition changes and/or phase transformation and therefore requires diffusion within grains. XRF equally captures composition changes within grains and grain boundaries but still requires a certain concentration change in the measurement spot. For this reason, longer-range diffusion along the grain boundaries may well be taking place, but it would not be readily captured by either XRD (because of the very small volume fraction affected) or micro-XRF (because of both the small volume and low concentration change).

### 3.2 XRD

XRD is by far the most widespread and established method to assess chemical compatibility between SOFC materials.^45−48^ Ideally, compatible materials do not give rise to secondary phases, even upon prolonged heating, so the XRD patterns of inert cathode/electrolyte annealed powders will not display any difference from the pristine phases. In the XRD patterns of the cubic BLF powders (space group *Pm-*3*m*), the peaks move slightly to higher angles with increasing La content (smaller lattice parameters, mainly caused by the smaller ionic radius of La^3+^ 1.36 Å compared to Ba^2+^ 1.61 Å).^49^

#### 3.2.1 BLF/GDC Couples

**Figure 1:**
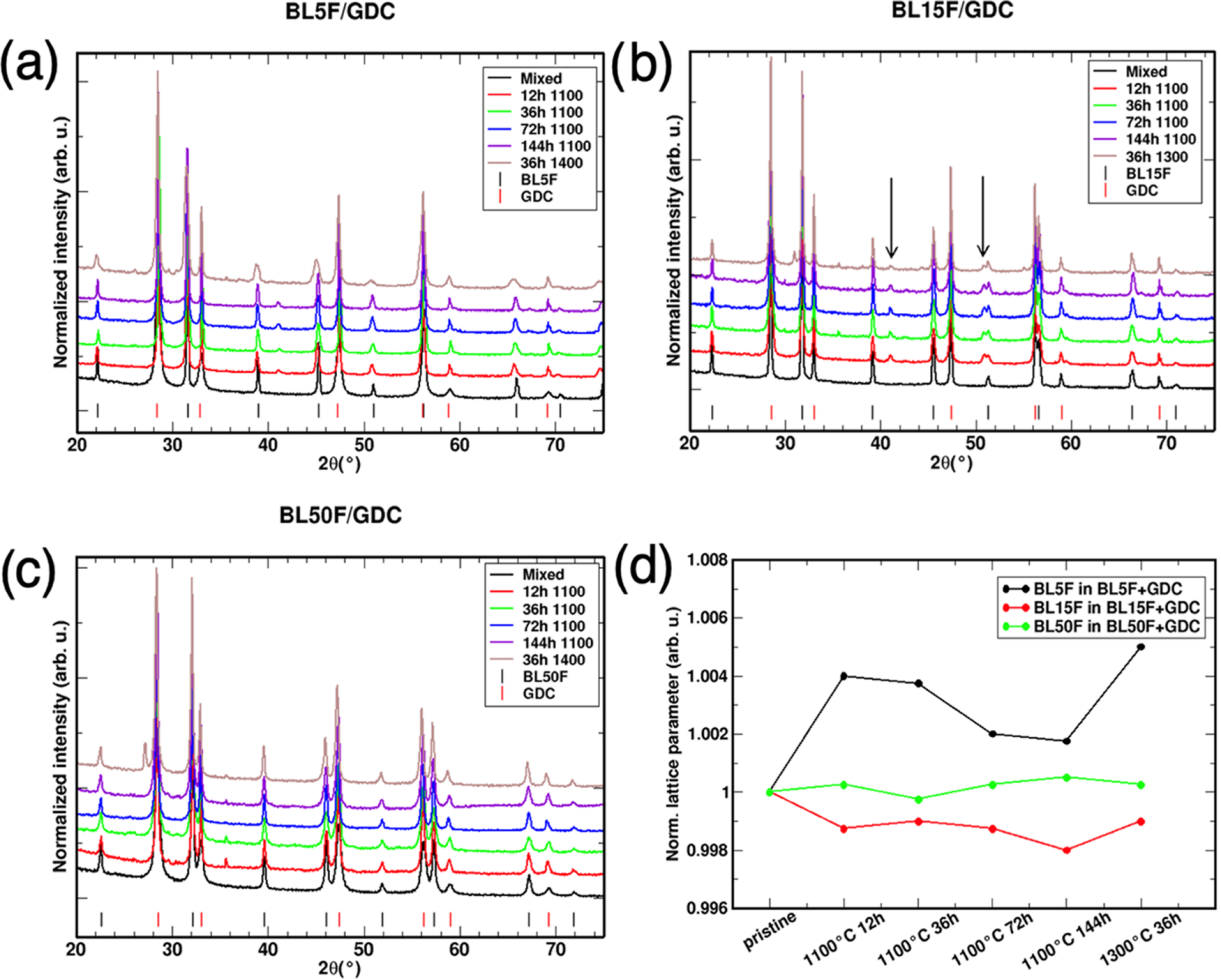
XRD patterns of the BLF/GDC samples. (a) BL5F/GDC; (b) BL15F/GDC; (c) BL50F/GDC; (d) evolution of the lattice parameter of BLF in contact with GDC as a function of treatment. The black arrows indicate the peak identification of BaCeO_3_.

The diffractograms of the annealed diffusion couples with GDC in Figure 1 demonstrate the appearance of secondary phases (Table 1). XRD patterns of BL5F/GDC and BL15F/GDC show similar features (Figure 1). The 12 h samples show the formation of a BaCeO_3_ secondary phase identified by (i) a small shoulder on the right side of the GDC (space group *Fm*3̅*m*) main peak (28.5°) and (ii) small peaks around 41 and 51° (Figure 1). In several samples, one more secondary phase is detected around 36°. Peak overlapping hinders an exact assignment, but tentatively this phase is assigned as structurally closely related γ-Fe_2_O_3_ or Fe_3_O_4_, and labeled as Fe_*x*_O_*y*_ in the following. BL15F/GDC shows an unassigned peak at 30° when it is treated for 36 h or more. When treated for 72 h or more, the BaCeO_3_ increases and Fe_*x*_O_*y*_ disappears. Treatment at 1300 °C resulted in a small amount of BaCeO_3_ for BLF15/GDC (Figure 1), which is absent for BL5F/GDC (Figure 1). In BL15F/GDC, one additional phase can tentatively be identified as hexagonal BaFeO_3_ from a peak at 31°: as shown with microspectroscopy below, the La/Ba value increases slightly at the boundary with GDC, and this imbalance probably also results in some BaFeO_3_ left behind.

**Table 1:** Summary of the Secondary Phases Observed for the BLF/GDC Couples^a^

BLF/GDC couple	BaCeO_3_	Fe*_x_*O*_y_*	BaFe_2_O_4_	unidentified phase
BL5F 12, 36, 72, 144 h	41° (4.7–5.2%)			
BL5F 36 h, 36 h (1300 °C)		36° (+)		
BL15F 12, 36, 72, 144 h, 36 h (1300 °C)	41°, 51° (3.3–5.4%)			
BL15F 36 h, 36 h (1300 °C)		36° (+)		
BL15F 36, 72, 144 h, 36 h (1300 °C)				29–31° (+)
BL50F 12, 36, 144 h, 36 h (1300 °C)		36° (+)		
BL50F 36 h (1300 °C)			27° (5.9%)	
BL50F 12, 36, 144 h				29–31° (+)

a Quantification is reported in parentheses, with either a weight fraction from Rietveld refinement when a single phase could be identified, or with a qualitative assessment using plus sign.

In BL50F/GDC, no peaks of a crystalline BaCeO_3_ phase are observed (Figure 1), which might be related to the cathode’s higher lanthanum and correspondingly lower barium content. After 12 h, the Fe_*x*_O_*y*_ peak at around 36° is visible. Moreover, between 29 and 31.3°, three peaks belonging to unidentified secondary phases are observed. No secondary phases are observed in BL50F/GDC 72 h. The iron oxide phase decreases in BL50F/GDC 144 h while the unknown secondary phases remain unchanged. In BL50F/GDC treated at 1300 °C, the iron oxide phase is still present but in a minimal amount. BaFe_2_O_4_ can be tentatively identified by a peak at 27°, again indicating some lanthanum diffusion into GDC at the highest annealing temperature.

The variation of the BLF lattice parameters is given in Table 2 and displayed in Figure 1, showing perceptible changes for BL5F and BL15F relative to those of the pristine materials (0.2–0.5%). However, there is no systematic increase with increasing annealing time at 1100 °C, indicating that the situation at the interfaces stabilizes already on a time scale of 12–36 h. The driving force for the reaction with GDC is still comparably small (Ba used for BaCeO_3_ formation has to be accommodated in the form of Ba vacancies in the BLF perovskite structure or complete decomposition of the BLF perovskite) such that no high degree of conversion occurs even for long annealing times. Overall, both BL5F and BL15F exhibit a significantly higher reactivity with GDC than BL50F. This may be related to two factors. (i) the Ba-rich compositions have overall a more basic character and thus are expected to have a higher reactivity with GDC (an oxide with slightly acidic character). (ii) BL5F and BL15F have Goldschmidt tolerance factors of *t* = 1.055 and 1.045, respectively, while BL50F has *t* = 1.015 (calculated using the averaged radii for Fe^3+^ and Fe^4+^ oxidation states).^49^ A larger deviation from the ideal *t* = 1 value means that the perovskite structure is less stable, and therefore more susceptible to react with another oxide such as GDC. BaCeO_3_ formation from reaction with GDC has also been observed for other alkali earth-rich perovskites with *t* > 1 such as Ba_0.5_Sr_0.5_Co_0.8_Fe_0.2_O_3−δ_.^50^ The importance of perovskite lattice stability as indicated by the tolerance factor shows up also when comparing the reactivities of the same set of La(Mn,Fe,Co)O_3−δ_ cathode materials with BaZrO_3_ (*t* = 1.004)^51^ and SrCeO_3_ (*t* = 0.885)^52^ electrolytes, where the latter shows a much higher reactivity. Similarly, BaCeO_3_ electrolytes (*t* = 0.938) were found to have a higher reactivity compared to BaZrO_3._^53^ Higher stability of BL50F notwithstanding, a small but significant lattice size increase is noticeable in GDC due to La^3+^ diffusion from BL50F (La^3+^ being 20% larger than host Ce^4+^).

**Table 2:** Lattice Parameters for BLF/GDC Couples

	BL5F/GDC		BL15F/GDC		BL50F/GDC	
	BL5F *a* (Å)	GDC *a* (Å)	BL15F *a* (Å)	GDC *a* (Å)	BL50F *a* (Å)	GDC *a* (Å)
pristine	4.003	5.424	3.985	5.424	3.937	5.424
12 h	4.019	5.424	3.980	5.425	3.938	5.431
36 h	4.018	5.425	3.981	5.425	3.936	5.427
72 h	4.011	5.424	3.980	5.426	3.938	5.435
144 h	4.010	5.424	3.977	5.425	3.939	5.428
36 h (1300 °C)	4.023	5.423	3.981	5.427	3.938	5.432

#### 3.2.2 BLF/BZY Couples

**Figure 2:**
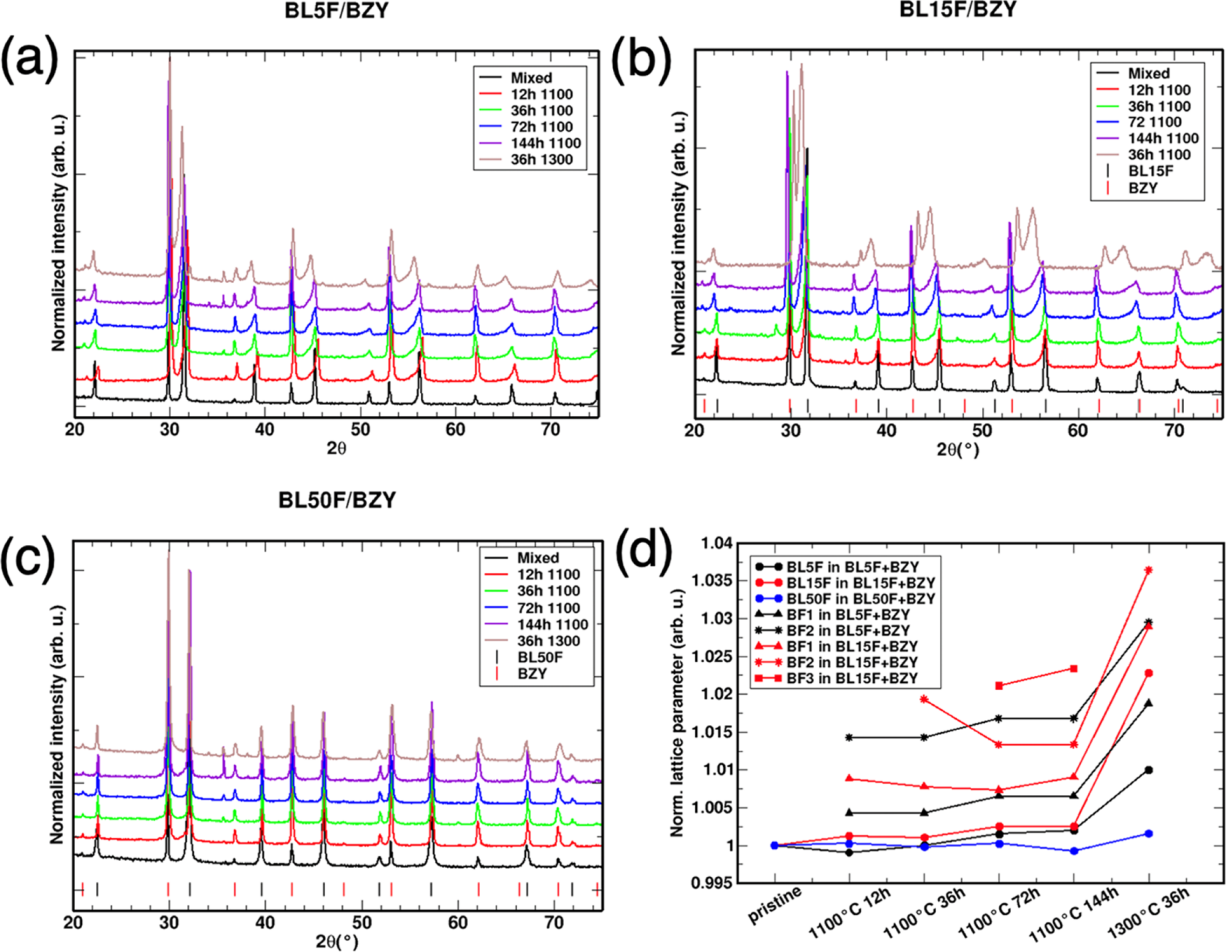
XRD patterns of the BLF/BZY samples. (a) BL5F/BZY; (b) BL15F/BZY; (c) BL50F/BZY; (d) evolution of the lattice parameter of BLF in contact with BZY as a function of treatment.

Interestingly, BLF powders show a markedly higher reactivity with the BZY perovskite than with the GDC fluorite electrolyte material. For this reason, each composition is worth discussing separately in the following. All of the XRD patterns and results of the annealed BLF/BZY couples are shown in Figure 2. Results are summarized in Tables 3 and 4.

**Table 3:** Summary of the Secondary Phases Observed for the BLF/BZY Couples^a^

BLF/BZY couple	Fe*_x_*O*_y_*	LaFeO_3_	La_2_Zr_2_O_7_	La_2_O_3_
BL5F 12, 36, 144 h, 36 h (1300 °C)	30–40° (++)			
BL5F 144 h, 36 h (1300 °C)		32° (1.1–1.8%)		
BL15F 12, 36, 72 h			27–30° (0.4–2.4%)	
BL50F 12, 36, 72, 144 h, 36 h (1300 °C)	36° (++)			29° (0.4–2.1%)
BL50F 36 h (1300 °C)	34° (+)			

a Quantification is reported in parentheses, with either a weight fraction from Rietveld refinement where a phase could be identified or with a qualitative assessment using plus sign.

**Table 4:** Lattice Parameters for the BLF/BZY Couples

	BZY, *a* (Å)	BL5F, *a* (Å)	BF1, *a* (Å)	BF2, *a* (Å)
pristine	4.220	4.003		
12 h	4.225	3.999	4.020	4.060
36 h	4.225	4.003	4.020	4.060
72 h	4.229	4.009	4.029	4.070
144 h	4.229	4.011	4.029	4.070
36 h, 1300 °C	4.211	4.043	4.078	4.121

	BZY, *a* (Å)	BL15F, *a* (Å)	BF1, *a* (Å)	BF2, *a* (Å)	BF3, *a* (Å)
pristine	4.220	3.985			
12 h	4.230	3.990	4.020		
36 h	4.226	3.989	4.016	4.062	
72 h	4.234	3.995	4.014	4.038	4.069
144 h	4.226	3.995	4.021	4.048	4.078
36 h, 1300 °C	4.197	4.076	4.100	4.130	

	BZY, *a* (Å)	BL50F, *a* (Å)	BF1, *a* (Å)
pristine	4.220	3.937	
12 h	4.227	3.938	
36 h	4.228	3.936	
72 h	4.226	3.938	
144 h	4.225	3.934	(*a* = 3.943, *b* = 3.953)
36 h, 1300 °C	4.218	3.943	

##### 3.2.2.1 BL5F/BZY

The main features in the annealed samples are the increased asymmetry of the BL5F peaks and the peaks appearing in the 30–40° range, all of which increase with longer treatments (Figure 2). After 12 h, two features appear: (i) has a small peak at 36°, probably due to Fe_*x*_O_*y*_; (ii) the BL5F peaks are skewed toward low angles. Such peak asymmetry is heuristically modeled with two additional perovskite phases labeled BF1 and BF2 having expanded cells with 4.02 and 4.06 Å (compared with pristine BL5F at 4.00 Å, Table 4). The pattern after 36 h is similar to 12 h, with a slight enlargement of the BZY cell and increased asymmetry of the BLF peaks. Small peaks at 36, 37.3, and 43.3° indicate Fe_3_O_4_ segregation in this sample. After 72 and 144 h, the lattice constants of all of the BLF phases further increase. The lattice constant of BZY also increased to 4.229 Å. A minor peak at 34° could not be attributed unambiguously. In the sample annealed at 1300 °C, one more secondary phase can be noticed from a new small peak around 32°, tentatively attributed to the orthorhombic perovskite LaFeO_3_, formed after La and Fe demixing from BL5F. More importantly, the BLF lattice parameters increase further (4.043 Å for BL5F, 4.078 Å for BF1, and 4.121 Å for BF2), while the cell size of BZY decreases slightly (4.211 Å). It is likely that given enough reaction time in contact, the BZY and BL5F would form a single cubic perovskite structure Ba(Zr,Fe,Y)O_3−δ_ with the final stoichiometry depending on the initial amounts of BL5F and BZY (a BaZr_0.88–*x*_Fe_*x*_Y_0.12_O_3−δ_ solid solution has been reported).^54^ BaFe_0.5_Zr_0.5_O_3_ has been described as a pseudotetragonal phase,^55,56^ and a cubic BaZr_1–*x*_Fe_*x*_O_3−δ_ (0 ≤ *x* ≤ 0.9) solid solution is reported in ref (57). The solid solution formation can be regarded as driven by configurational entropy, as well as by acid–base interactions between a relatively acidic BLF perovskite and a basic perovskite BZY, due to the significantly smaller ion radius of Fe^3+^/Fe^4+^ (0.645–0.585 Å) compared to that of Zr^4+^/Y^3+^ (0.72–0.9 Å). The same considerations do not apply when BLF is in contact with BaCeO_3_, since the lattice size mismatch is much larger, resulting in an extended miscibility gap (as witnessed by a mutual solubility of BaCeO_3_ and BaFeO_3_ of only 15%^58^).

##### 3.2.2.2 BL15F/BZY

The BL15F/BZY samples follow a trend similar to that of BL5F/BZY (Figure 2). The peaks of BL15F show the same asymmetry toward lower angles as seen in BL5F/BZY, with stronger relative changes of the BLF lattice parameters (Figure 2 and Table 4, one to three BLF phases were added). The lattice parameters of BZY follow a more erratic trend. At 1300 °C, the BLF and BZY peaks almost merge; here again, a complete solid solution could be expected for prolonged annealing. The low-intensity peaks at 27 and 33° are attributed to the formation of La_2_Zr_2_O_7_: its amount increases from 12 to 36 h, then decreases at 72 h, and disappears at 144 h. The formation of La_2_Zr_2_O_7_ is probably related to the higher La content of BL15F compared to BL5F; the decrease at a longer time and higher temperature might result from the increasing formation of the (Ba,La)(Fe,Zr)O_3_ solid solution phase.

##### 3.2.2.3 BL50F/BZY

In BL50F/BZY, at variance with the previous compositions, there is no skewness in the BLF peaks but some reactivity between the materials can still be recognized from the appearance of new peaks (Figure 2). At 12–72 h, two different secondary phases are visible by two small peaks: (i) around 29°, identified as La_2_O_3_ demixing from BL50F; (ii) around 36°, previously identified as Fe_*x*_O_*y*_, systematically increasing with annealing time. The 144 h sample shows two new features: (i) the Fe_*x*_O_*y*_ phase increases, but just using the Fe_3_O_4_ phase to model the peak does not yield a satisfactory fit in this case, and a mixture of hematite and magnetite is likely present; (ii) the skewness of BL50F is most evident around the peak at 32° 2θ. A new tetragonal BaFeO_3_ is introduced to model this skewness, with lattice parameters *a* = 3.943 Å and *c* = 3.953 Å. The sample annealed at 1300 °C shows the Fe_*x*_O_*y*_ phase with a lower intensity probably due to further cation diffusion. In this sample, a new phase appears (new peak at ∼34° 2θ), but it was not possible to assign it to a specific crystal structure.

Overall, BL50F shows much less reactivity with BZY compared to BL5F and BL15F. Such inherent stability may come from structural consideration in the first place, i.e., higher stability of the pristine perovskite structure, as BL50F deviates very little from the ideal Goldschmidt tolerance factor. Also, the high La^3+^ content makes the combination of all cations into a single (Ba,La)(Zr,Fe,Y)O_3_ phase less favorable, as the lattice parameter mismatch between the hypothetical LaFeO_3_ (pseudocubic lattice parameter 3.93 Å) and BZY (4.22 Å) end members would be unfavorably large. For the BLF/BZY couples, these structural factors obviously dominate over acid–base interactions, as the basicity difference of BL50F (the least basic ferrite owing to low Ba content) to the basic electrolyte BZY is larger than that for BL5F and BL15F.

### 3.3 X-ray Microspectroscopy

Micro-X-ray fluorescence (XRF) elemental mapping was performed in order to investigate cation interdiffusion after thermal aging at 1100 °C for 72 h. The resulting concentration profiles were obtained vertically (across the cathode and the electrolyte) by integrating the elemental concentrations along the interface. Then, micro-X-ray absorption near-edge structure (micro-XANES) spectra at the Fe or Gd absorption edges were acquired in several selected spots to obtain insights about the coordination of cations as well as their oxidation states.

#### 3.3.1 BLF/GDC Couples

##### 3.3.1.1 BL15F/GDC

**Figure 3:**
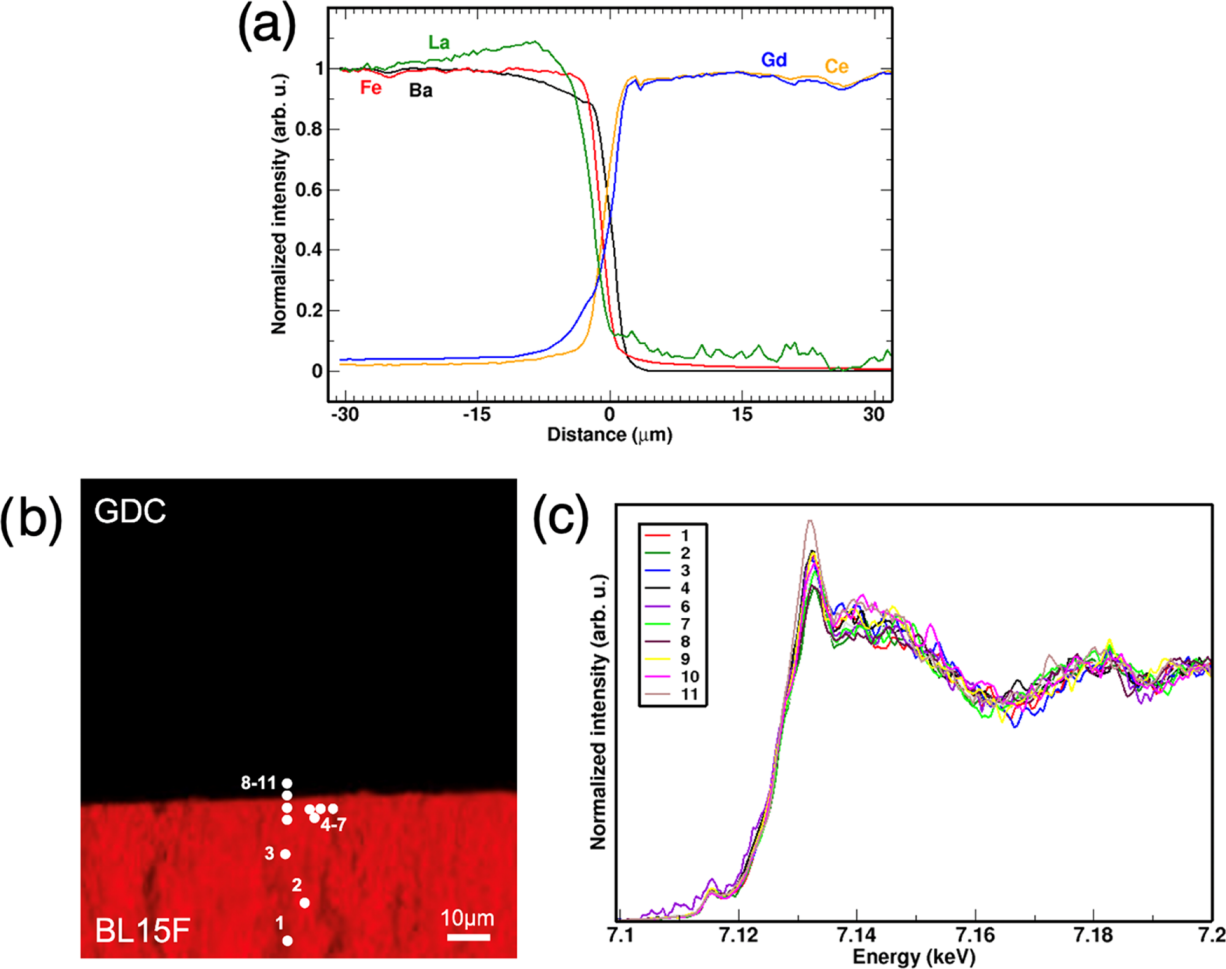
BL15F/GDC 72 h 1100 °C. (a) XRF cation concentration profiles of barium, lanthanum, iron, gadolinium, cerium. (b) Micro-XRF concentration map of iron; (c) Fe *K*-edge micro-XANES spectra measured at different points shown in the map: the black arrow marks the evolution of spectra from point 1 to 14.

No evident reaction features (i.e., formation of secondary phases) are observed at the cathode/electrolyte interface of the BL15F/GDC couple after 72 h of thermal aging at 1100 °C (Figure 3). The concentration profiles of lanthanum and barium show opposite relative trends: lanthanum increases rapidly toward the interface region for approximately 20 μm, while barium decreases (absolute changes shown in the bulk-rescaled profiles in the SI). The iron content shows a very sharp and steep profile indicating low cation diffusion through the cathode/electrolyte couple. The diffusion profiles of gadolinium and cerium are quite similar directly at the interface. Gadolinium shows an additional tailing deeper into BLF, indicating a higher mobility of Gd^3+^ than Ce^4+^, likely because of its lower charge. A Ba excess (and La depletion) is visible directly at the interface, which agrees well with the BaCeO_3_ peak observed in XRD.

The micro-XANES spectra acquired at the Fe K-edge show that the overall shape of the absorption edge peaks remains fairly unchanged in the different spots examined as the XANES white line changes intensity (Figure 3a). This suggests that iron is subject to changes of its overall chemical environment, but still preserving its octahedral coordination in the B-site of a perovskite environment in BLF with oxidation states between +3 and +4, and (Ce,Gd)FeO_3_ in the GDC region,^53^ without being involved in cation substitution (e.g., for Ce^4+^ in GDC).

##### 3.3.1.2 BL50F/GDC

**Figure 4:**
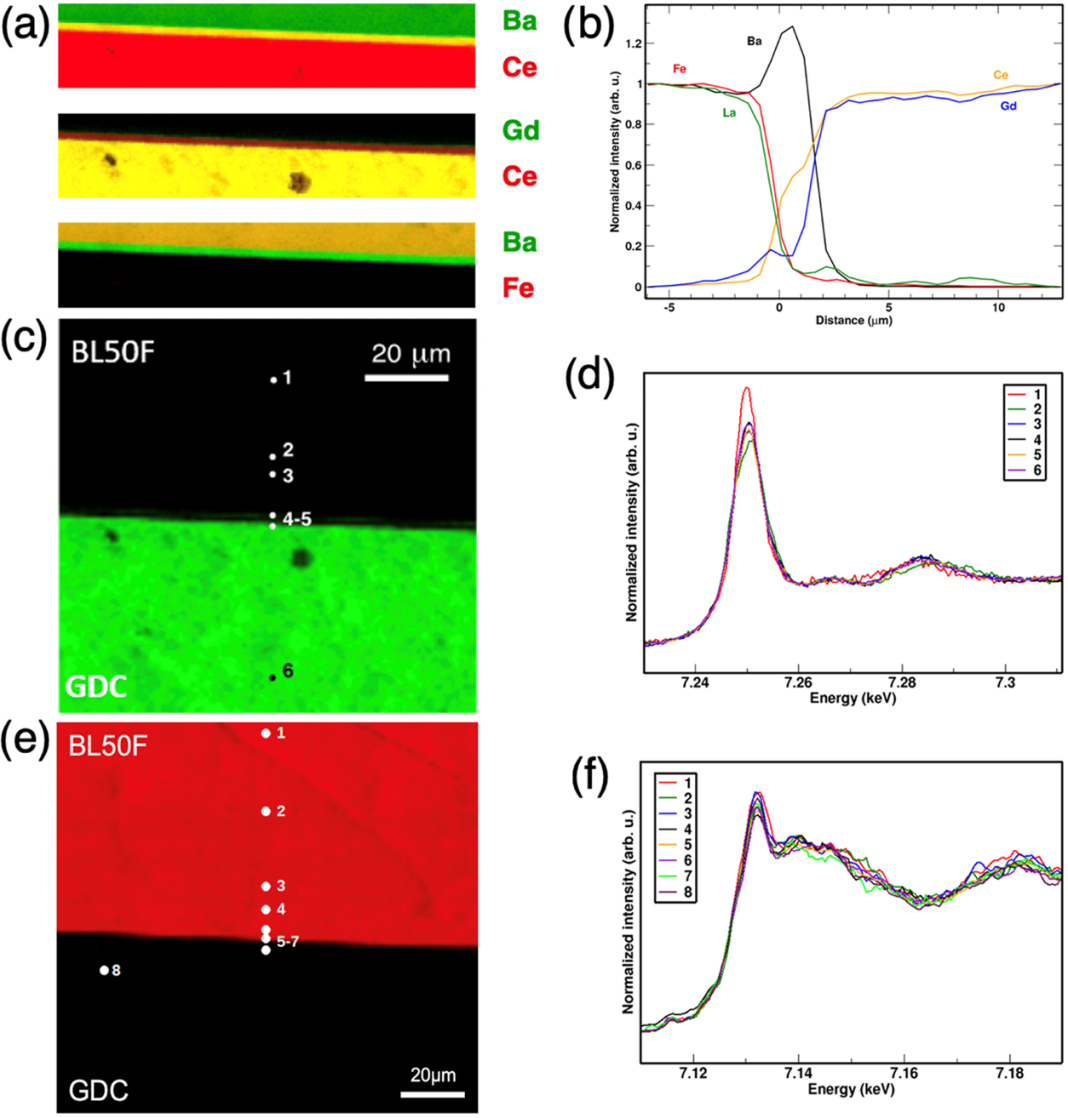
BL50F/GDC. (a) Magnification of the interface (20 μm across): superimposed XRF concentration maps of: (top) Ba and Ce; (middle) Gd and Ce; and (bottom) Ba and Fe. Green and red superimposition results in yellow. (b) XRF concentration profiles of barium, lanthanum, iron, gadolinium, and cerium. (c) Concentration map of gadolinium (green). (d) Gd L_3_-edge micro-XANES spectra measured at different points shown in the map. (e) Concentration map of iron (red) and (f) Fe K-edge micro-XANES spectra measured at different points shown in the map.

The micro-XRF maps and concentration profiles of the BL50F/GDC couple are listed in Figure 4. Iron does not show strong evidence of long-range cation diffusion as its concentration remains constant at its highest value before decreasing at the interface. Lanthanum, shows a similar behavior, except for a low but significant incorporation in GDC which corroborates the earlier observation of an enlarged lattice parameter. Similarly, cerium and gadolinium concentrations remain constant before declining toward the cathode region. Similar to BL15F/GDC, gadolinium exhibits a longer tailing into the GDC compared to cerium. A thin reactivity zone at the interface (5 μm wide) reveals a certain cation interdiffusion between the cathode and electrolyte as displayed in the elemental maps (Figure 4a) and in the concentration profiles (Figure 4b). A sharp accumulation of barium can be observed at the interface/electrolyte boundary followed by an abrupt drop toward the electrolyte.

As for BL15F/GDC, the Ba profile extends deeper into the GDC than does the Fe profile, resulting in a Ba- and Ce-rich region that can again be tentatively identified as Gd-doped BaCeO_3_. The fact that this region is marked by a clear Ba/La dissimilarity, with a Ba concentration increase, can be explained by the fact that La^3+^ cannot be incorporated in the BaCeO_3_ A-site. A complete comparative representation of profiles BLF15F and BL50F is given in the SI. This sample demonstrates that it is helpful to investigate the evolution of the interface with complementary methods. The element-selective and space-resolved micro-XRF map elucidates that a layer with a composition close to barium cerate is formed, although it apparently has not crystallized well enough to show up as peaks in the XRD traces.

Micro-XANES spectra recorded at the Fe K-edge and Gd L_3_-edge are reported in Figure 4d–f. Each spot for both absorption spectra is measured inside BLF, except for the last one, which is in the GDC region. The shape of the Fe K-edge is similar to that acquired in the BL15F/GDC couple, and the same considerations hold. On the other hand, the analysis of Gd L_3_-edge provides evidence of some other compounds: the spectrum for point 1 presents three features (increase of the edge transition, flattening of the 7.26 keV and red shift of the feature at 7.28 keV) that are typical of perovskite A-site shared with a divalent cation, i.e., (Ba,Gd)FeO_3_; the spectrum of point 2 feature a definite broadening of the main edge that is typical of Gd_2_O_3_; the other spectra (points 3–6) confirm Gd^3+^ in the fluorite lattice of GDC.^59,60^

#### 3.3.2 BLF/BZY Couples

##### 3.3.2.1 BL15F/BZY

**Figure 5:**
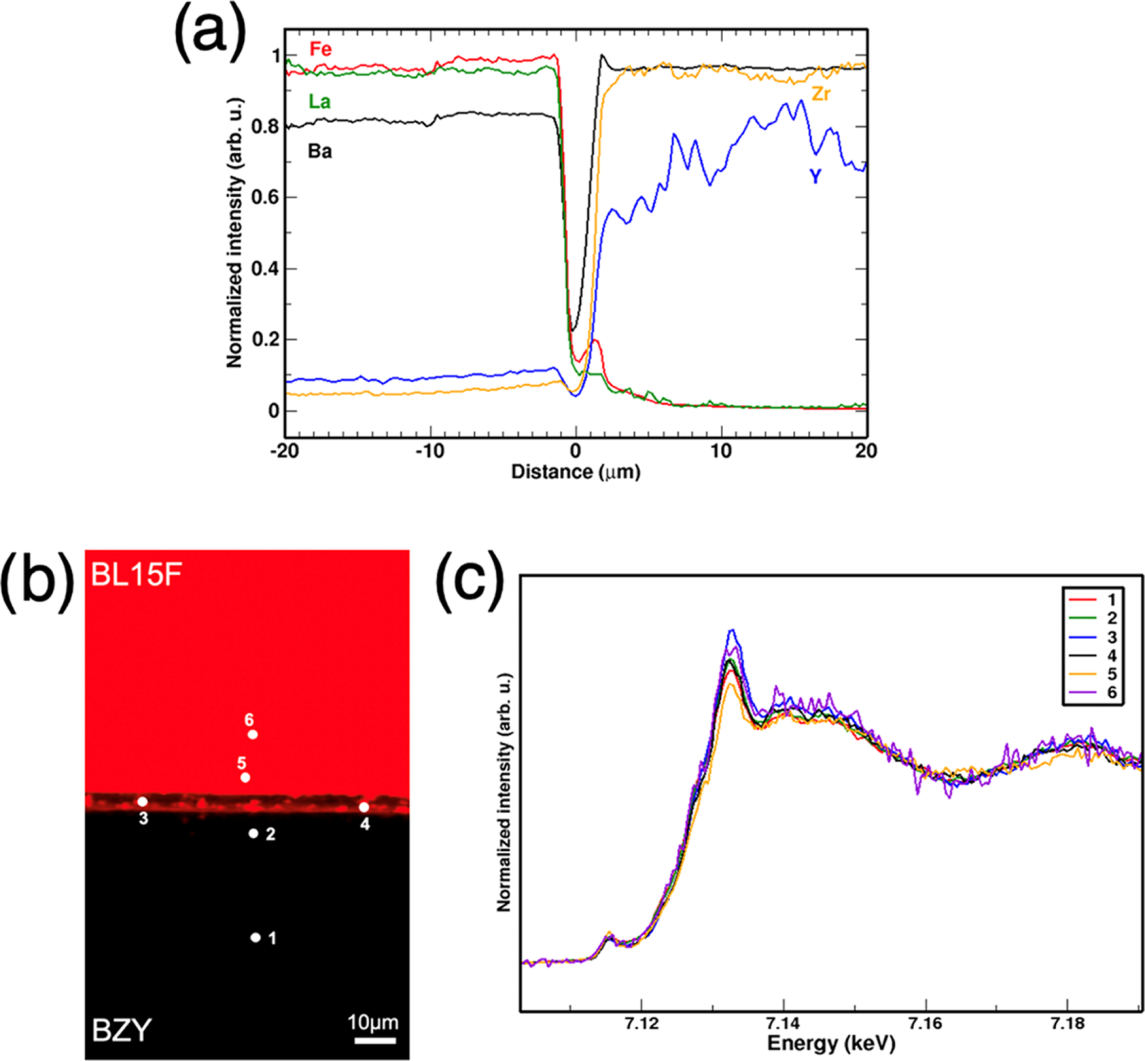
BL15F/BZY. (a) XRF concentration profiles of barium, zirconium, yttrium, lanthanum, and iron. (b) XRF concentration map of iron (red). (c) Fe *K*-edge micro-XANES spectra measured at different points.

The micro-XRF maps and cation profiles of the cathode/electrolyte interface of the BL15F/BZY couple after 72 h of thermal aging (Figure 5) show a shorter diffusion length for cations compared to the BLF/GDC samples. An earlier study on a BaZrO_3_/LaFeO_3_ diffusion couple also found very limited interdiffusion, although the presence of La^3+^ limits the mutual solubility of the two compounds.^51^ It is likely that the absence of cerium is mostly responsible for the markedly lower reactivity at the interphase, as BaCeO_3_-based electrolytes were found to readily react with a number of different electrode materials in a broad XRD survey while BaZrO_3_ did not.^53^ The intensity dip in the BLF15 phase close to the interface (cf. dark region above the interface in Figure 5b) is due to delamination that likely occurred during cooling since smooth diffusion profiles are visible beyond the interface on both sides. Lanthanum and iron are slightly enriched on the BZY side, close to the interface zone (about 2 μm), and the Fe profile decays over an additional 3 μm within the BZY. In the comparison to XRD (Figure 2b, with pronounced BLF15 peak asymmetry), one should consider that in the XRD samples with a grain size in the few micrometers range this interdiffusion zone represents a large volume fraction of the overall samples, and thus the Fe/Zr interdiffusion between BLF15 and BZY appears to be more prominent there. Yttrium shows micron-scale relative oscillations in its concentration, before decreasing close to the interface, and showing a small diffusion in the cathode region. The images in the SI show that the BZY phase has occasional porosity/inhomogeneity, which also contributes to XRF intensity fluctuations (similar for the BZY of the BZY/BLF50 couple).

The micro-XANES spectra acquired at the Fe *K*-edge are reported (Figure 5c). Also, in this case, the overall shape of the absorption edge is largely unmodified, indicating that iron maintains its position in the perovskite B-site on both cathode and electrolyte sides. All changes in the pre-edge and main edge features depend on the A-site (Ba^2+^/La^3+^) average valence, as well as on the possible Zr^4+^ content of the B-site.

##### 3.3.2.2 BL50F/BZY

**Figure 6:**
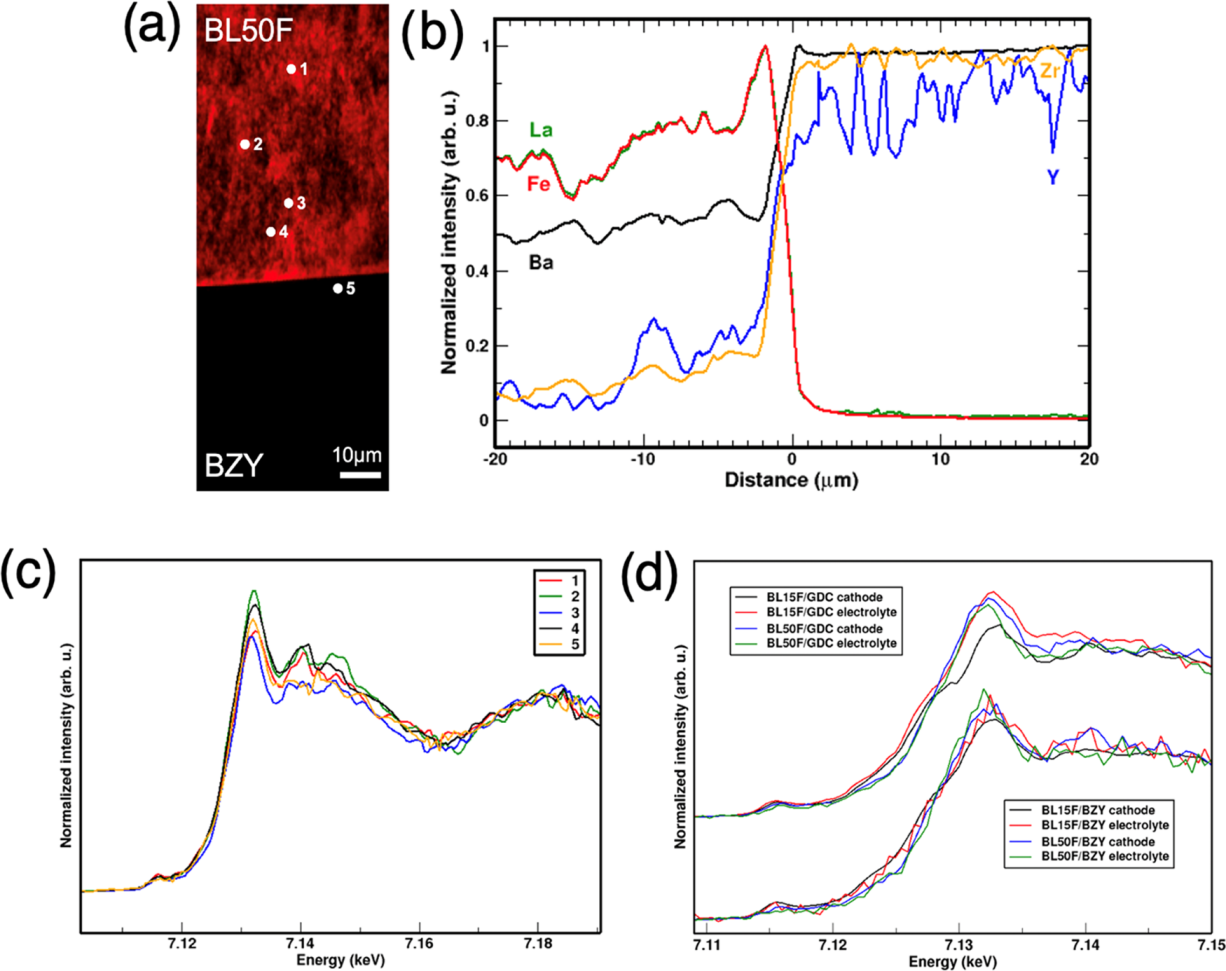
BL50F/BZY at the Fe K-edge. (a) XRF concentration map of iron (red) and Fe K-edge micro-XANES spectra measured at different points. (b) Fe *K*-edge micro-XANES spectra measured at different points. (c) Concentration profiles of barium (black), zirconium (yellow), yttrium (blue), lanthanum (green), and iron (red). (d) Comparison of Fe *K*-edge micro-XANES spectra of all samples.

Although a reactivity zone is not clearly separated from the two parent phases, the BL50F/BZY couple shows a significant cation accumulation at the cathode/electrolyte interface, as shown in Figure 6. As seen in the BL15F/BZY couple, the concentration of most cations in the electrolyte shows inhomogeneity to some extent, even more evident in the BL50F phase. In addition, lanthanum and iron profiles are now characterized by a sharp accumulation around 3 μm from the interface, probably due to iron oxides and some LaFeO_3_ (as La and Fe are completely correlated). Yttrium and zirconium also show inhomogeneous cation diffusion, as indicated by the presence of accumulation patches of various sizes within the cathode region visible in the fluorescence map (see the SI). In the micro-XANES spectra acquired at the Fe K-edge and reported in Figure 6, the overall shape typical of a perovskite environment is always maintained, although with a certain yet unsystematic variance which is uncorrelated with the position with respect to the interface. The significant inhomogeneity of the Ba/La ratio at the microscopic level (see the SI), and of the (Fe^3+^/Fe^4+^) valence as a consequence, is behind such variations.

As a conclusive remark, Figure 6 also sums up the fate of iron in the parent cathode phase and on the electrolyte side after diffusion in the four couples. As expected, by comparison of the edge shape, the main systematic effect occurs in the BL15F/GDC sample. On the other hand, both BLF/BZY couples do not show a systematic variation in the spectra after diffusion, which exemplifies the crystal-chemical closeness of the two Ba-based perovskite lattices.

## 4 Conclusions

The chemical and structural compatibility of Ba_1–*x*_La_*x*_FeO_3−δ_ (*x* = 0.05–0.5) electrode materials with oxide-ion conducting Ce_0.8_Gd_0.2_O_2-δ_ and proton-conducting BaZr_0.825_Y_0.175_O_3−δ_ electrolytes was investigated on diffusion couples thermally aged at 1100–1300 °C. Complementary techniques of X-ray diffraction (probing large volumes in the bulk and yielding crystallographic information) and X-ray microspectroscopy (chemical and structural information on a micrometer length scale) were employed.

We found that the compatibility of (Ba,La)FeO_3−δ_ with both electrolytes is strongly dependent on the amount of lanthanum. With GDC, the majority of impurity phases formed are Fe_*x*_O_*y*_ and Gd-doped BaCeO_3_. Ba_0.95_La_0.05_FeO_3−δ_ and Ba_0.85_La_0.15_FeO_3−δ_ both show a higher reactivity than Ba_0.5_La_0.5_FeO_3−δ_, which is attributed to their larger deviation of the Goldschmidt factor from unity, suggesting a lower stability of the perovskite structure. This lower stability of the initial cathode phase implies a larger enthalpic driving force for interfacial reaction, which leads to more stable new phases, such as BaCeO_3_, or decreases the A/B cation size mismatch by interdiffusion. In particular, a high reactivity is found with the BZY electrolyte. In addition to small amounts of crystalline Fe_*x*_O_*y*_, La_2_O_3_, and La_2_Zr_2_O_7_ being formed at the interface, significant interdiffusion is observed for Ba_0.95_La_0.05_FeO_3−δ_ and Ba_0.85_La_0.15_FeO_3−δ_, resulting in a (Ba,La)(Fe,Zr,Y)O_3−δ_ perovskite solid solution.

For both GDC and BZY electrolytes, the majority of interfacial phases are expected not to be extremely detrimental for cell operation: Gd-doped BaCeO_3_ formed with GDC is still an oxygen ion conductor and proton/hole mixed-conducting (Ba,La)(Fe,Zr,Y)O_3−δ_ may serve as a smooth transition from electrolyte to electrode. At operating temperature for intermediate temperature fuel/electrolyzer cells in the range of ≤600 °C, cation diffusion is sufficiently slow such that these layers will not penetrate through the electrolyte. The present investigation indicates that an increased La content in Ba_1*–x*_La_*x*_FeO_3−δ_ (smaller deviation of the Goldschmidt factor) decreases the interfacial reaction with both electrolytes. Applying oversized B-site dopants such as Zn^2+^ or Y^3+^ is a further strategy to increase the Goldschmidt factor. In conclusion, this investigation provides a further understanding of the formed impurity phases and potential degradation mechanisms, providing tools to understand and design better-performing devices.

## Supporting Information Available

Bulk and GB cation diffusion coefficients from the literature; complete micro-XRF maps and concentration profiles; powder XRD Rietveld refinements; and SEM images (PDF)
