# Supplementary material for: Interface Diffusion and Compatibility of (Ba,La)FeO3−δ Perovskite Electrodes in Contact with Barium Zirconate and Ceria
Source: ACS Appl Mater Interfaces. 2023 Oct 20;15(43):50225–36. doi: 10.1021/acsami.3c13013 (PMC10623510; doi:10.1021/acsami.3c13013)
Supplement: Supplementary file 1 — am3c13013_si_001.pdf [file am3c13013_si_001.pdf]

Interface diffusion and compatibility of (Ba,La)$FeO_{3-\delta}$ perovskite electrodes in contact with barium zirconate and ceria

Alessandro Chiara[1], Giulia Raimondi[2], Rotraut Merkle[2], Joachim Maier[2], Claudio Ventura Bordenca[1], Candida Pipitone[1], Alessandro Longo[3,4], and Francesco Giannici[1,*]

[1] Dipartimento di Fisica e Chimica, Università di Palermo, 90128 Palermo, Italy

[2] Max Planck Institute for Solid State Research, 70569 Stuttgart, Germany

[3] Istituto per lo Studio dei Materiali Nanostrutturati (ISMN)-CNR, UOS Palermo, 90146 Palermo, Italy

[4] European Synchrotron Radiation Facility, 38000 Grenoble, France

* corresponding author: francesco.giannici@unipa.it

SUPPORTING INFORMATION

Table S1. Samples, annealing time and temperature, and analytical methods used.

Sample	XRD	XAS
**BL5F + GDC**	Pristine, 12h, 36h, 72h, 144h (1100°C), 36h (1300°C)	
**BL15F + GDC**	Pristine, 12h, 36h, 72h, 144h (1100°C), 36h (1300°C)	72h
**BL50F + GDC**	Pristine, 12h, 36h, 72h, 144h (1100°C), 36h (1300°C)	72h
**BL5F + BZY**	Pristine, 12h, 36h, 72h, 144h (1100°C), 36h (1300°C)	
**BL15F + BZY**	Pristine, 12h, 36h, 72h, 144h (1100°C), 36h (1300°C)	72h
**BL50F + BZY**	Pristine, 12h, 36h, 72h, 144h (1100°C), 36h (1300°C)	72h

Cation diffusion coefficients from literature

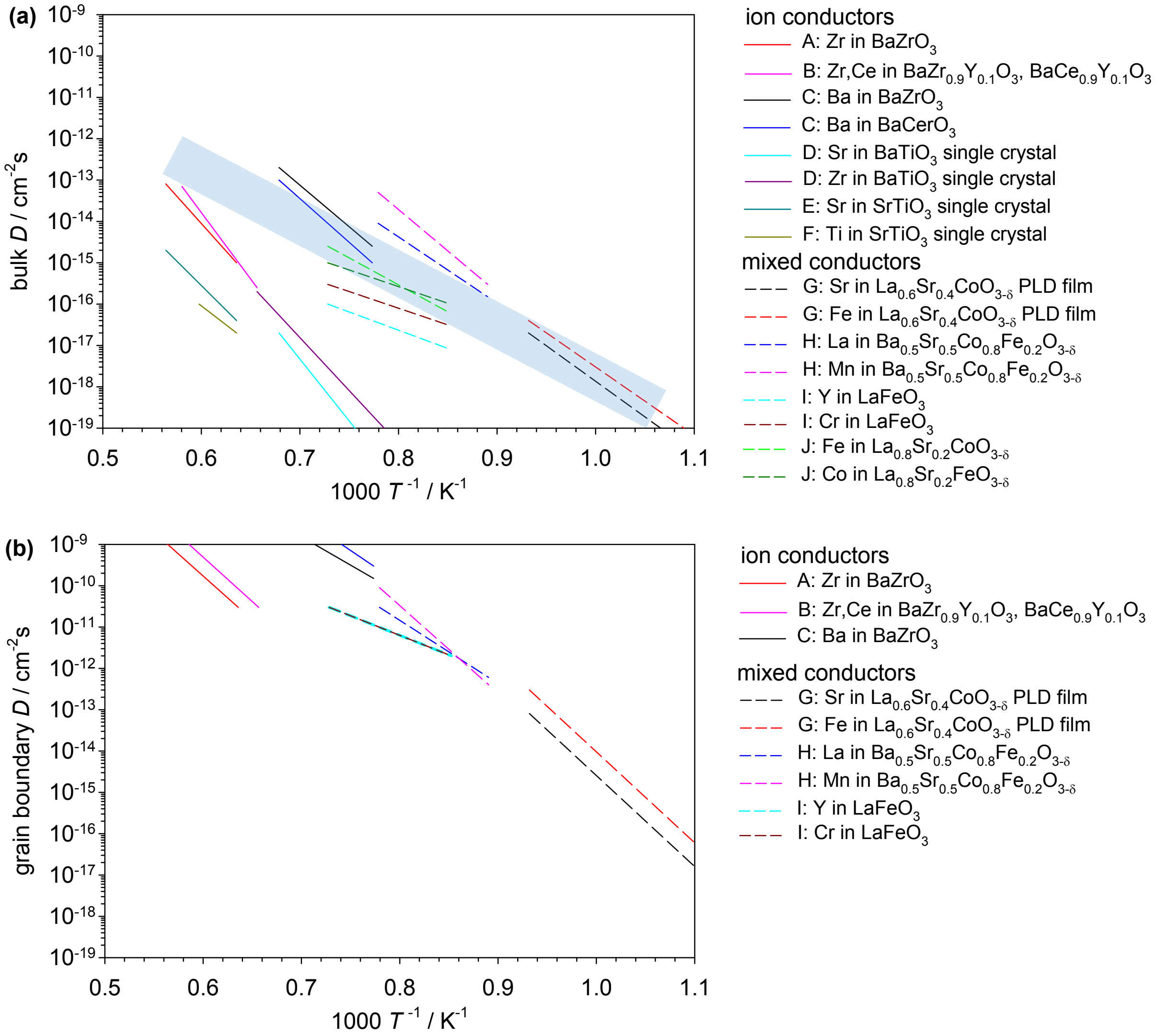

Figure S1: (a) Bulk and (b) grain boundary cation diffusion coefficients in various perovskites. The grain boundary diffusivities shown here are typically calculated as local diffusivity in a 1 nm wide region comprising the boundary. The blue shaded region indicates an average over the bulk diffusivities of representative perovskite compositions in an extended temperature range. All diffusion coefficients are self/tracer diffusion coefficients; if suitable isotopes are unavailable, chemically similar “impurity cations” were used. The details are specified below, typically the tracer is applied as thin film source. B and J are interdiffusion between large ceramic pellets.

Data sources for Figure S1:

A: Sazinas, R.; Sakaguchi, I.; Hasle, I.; Polfus, J. M.; Haugsrud, R.; Einarsrud, M. A.; Grande, T. *Phys. Chem. Chem. Phys.* **2017**, 19, 21878-21886. ^{134}Ba and ^{96}Zr tracers, applied as thin film.

B: Hasle, I.; Waldow, S. P.; Gries, U. N.; De Souza, R. A.; Vollestad, E.; Haugsrud, R. *J. Mater. Chem. A* **2021**, 9, 21142-21150. Interdiffusion between $BaZr_{0.9}Y_{0.1}O_{2.95}$ and $BaZr_{0.9}Y_{0.1}O_{2.95}$ ceramics.

C: Sazinas, R.; Sakaguchi, I.; Einarsrud, M. A.; Grande, T. *AIP Adv.* **2017**, 7, 115204. ^{134}Ba tracer, applied as thin film.

D: Koerfer, S.; De Souza, R. A.; Yoo, H. I.; Martin, M. *Solid State Sci.* **2008**, 10, 725-734. Sr and Zr "impurity cations", applied as thin film.

E: Gömann, K.; Borchardt, G.; Schulz, M.; Gömann, A.; Maus-Friedrichs, W.; Lesage, B.; Kaitasov, O.; Hoffmann-Eifert, S.; Schneller, T. *Phys. Chem. Chem. Phys.* **2005**, 7, 2053-2060. ^{87}Sr tracer, applied by ion implantation.

F: Gömann, K.; Borchardt, G.; Gunhold, A.; Maus-Friedrichs, W.; Baumann, H. *Phys. Chem. Chem. Phys.* **2004**, 6, 3639-3644. $^{49,50}Ti$ tracer, applied by ion implantation.

G: Kubicek, M.; Rupp, G. M.; Huber, S.; Penn, A.; Opitz, A. K.; Bernardi, J.; Stöger-Pollach, M.; Hutter, H.; Fleig, J. *Phys. Chem. Chem. Phys.* **2014**, 16, 2715-2726. ^{86}Sr tracer, Sm and Fe "impurity cations" applied as thin film.

H: Harvey, S. P.; De Souza, R. A.; Martin, M. *Energy Environ. Sci.* **2012**, 5, 5803-5813. La and Mn "impurity cations", applied as thin film.

I: Waernhus, I.; Sakai, N.; Yokokawa, H.; Grande, T.; Einarsrud, M. A.; Wiik, K. *Solid State Ionics* **2007**, 178, 907-914. Y and Cr "impurity cations", applied as thin film.

J: Kishimoto, H., Sakai, N.; Horita, T.; Yamaji, K.; Brito, M. E.; Yokokawa, H. *Solid State Ionics* **2007**, 178, 1317-1325. Interdiffusion between $LaFeO_{3-\delta}$ and $LaCoO_{3-\delta}$ ceramics.

Despite perceptible scatter, some trends can be recognized for cation diffusivities in perovskites (typically acceptor-doped): (i) there is no systematic difference between ion-conducting electrolyte-type and mixed-conducting perovskites; (ii) the diffusivity values for A-site and B-site cations are roughly comparable, indicating a transport mechanism involving common defects; (iii) the cation diffusivities have significantly higher activation energies (3-5 eV) compared to oxygen vacancies (0.7-1 eV); (iv) the titanate perovskites with rather small lattice parameters seem to be systematic outliers in the plot for bulk diffusivity, thus they are not considered for the estimation of diffusion lengths; (v) the local grain boundary diffusivities are several orders of magnitude higher than bulk values. All considered, the effective cation diffusion coefficient of a polycrystalline sample depends on the grain size (i.e. volume fraction of the grain boundary zone).

X-ray microspectroscopy

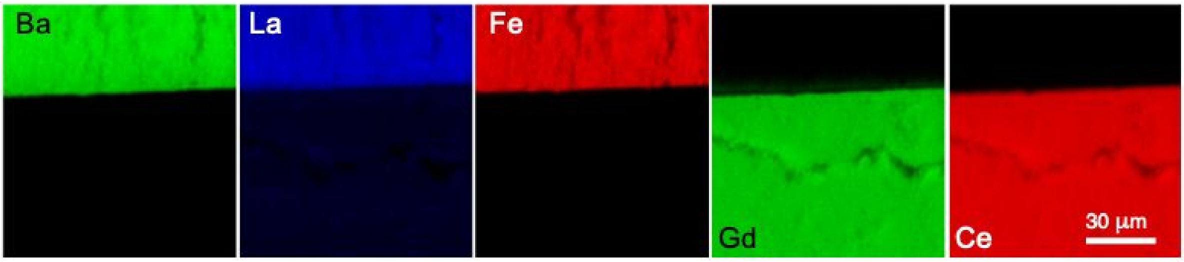

Figure S2 – BL15F/GDC 72h at the Fe K-edge. Left to right: XRF concentration maps of barium (green), lanthanum (blue), iron (red), gadolinium (green) and cerium (red).

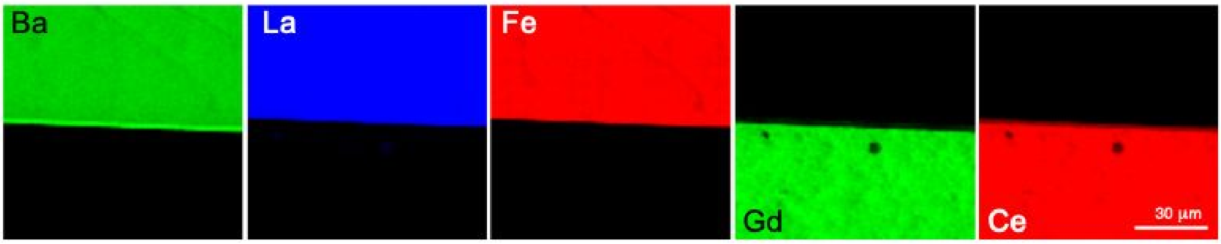

Figure S3 – BL50F/GDC 72h at the Fe K-edge. Left to right: XRF concentration maps of barium (green), lanthanum (blue), iron (red), gadolinium (green) and cerium (red).

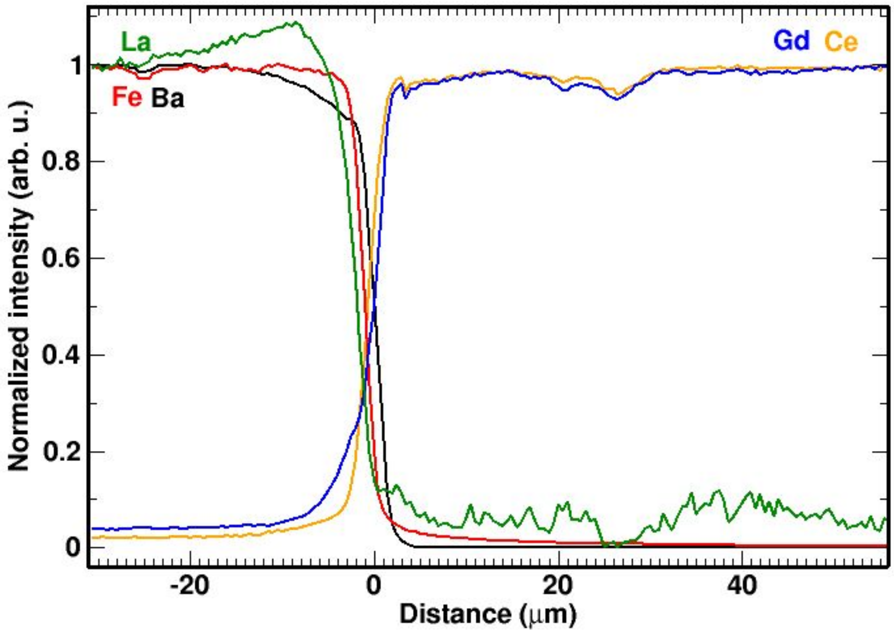

Figure S4 – BL15F/GDC 72h at the Fe K-edge. Concentration profiles of barium (black), lanthanum (yellow), iron (green), gadolinium (blue), cerium (red).

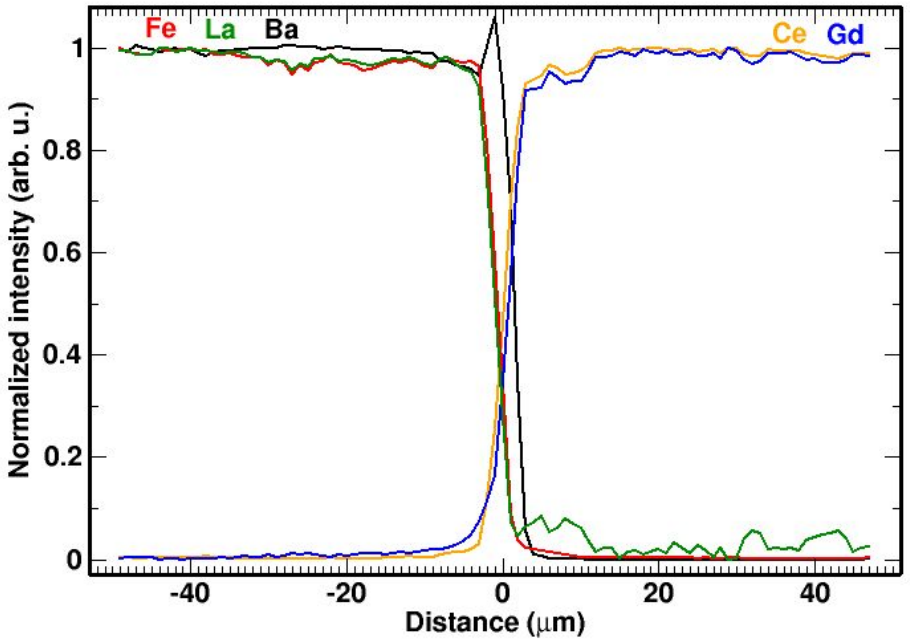

Figure S5 – BL50F/GDC 72h at the Fe K-edge. Concentration profiles of barium (black), lanthanum (yellow), iron (green), gadolinium (blue), cerium (red).

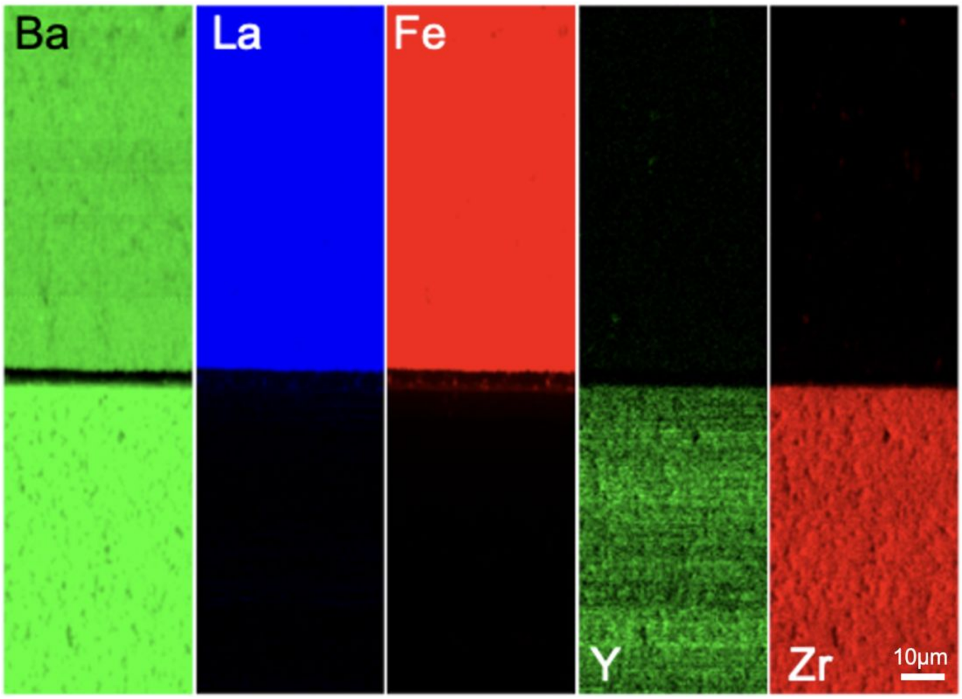

Figure S6 – BL15F/BZY. Left to right: XRF concentration maps of barium (green), lanthanum (blue), iron (red), yttrium (green) and zirconium (red).

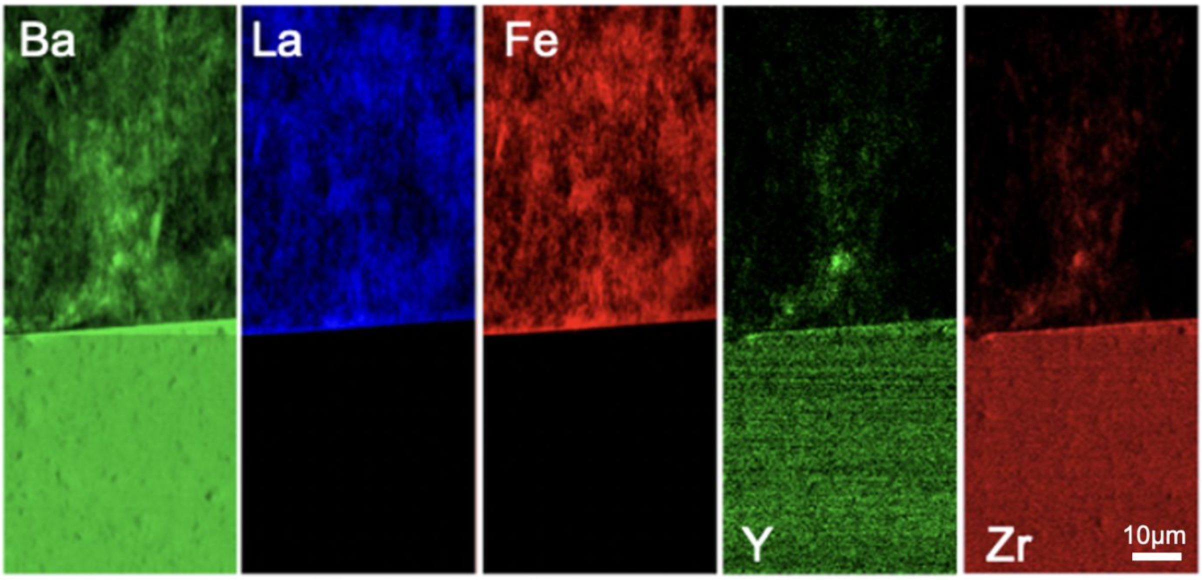

Figure S7 – BL50F/BZY. Left to right: XRF concentration maps of barium (red), iron (red), lanthanum (green), yttrium (green) and zirconium (blue).

As shown in elemental maps S7 and S8, and in the corresponding profiles S11 and S12, BZY shows some inhomogeneity in the Zr/Y ratio at the micrometer scale, and occasional porosity. BL50F, on the other hand, features extensive Ba/La inhomogeneity (possibly driven by Zr/Y interdiffusion) while BL15F is remarkably stable in comparison. Figure S13 further illustrates the imbalance between A-site and B-site cations in BL50F close to the interface.

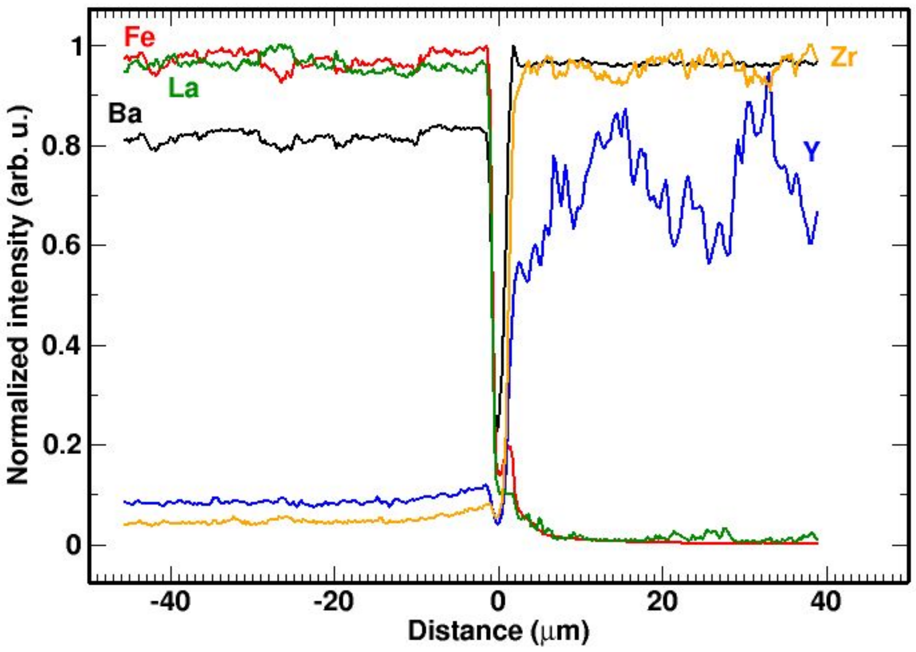

Figure S8 - BL15F/BZY. Concentration profiles of barium, zirconium, yttrium, lanthanum and iron.

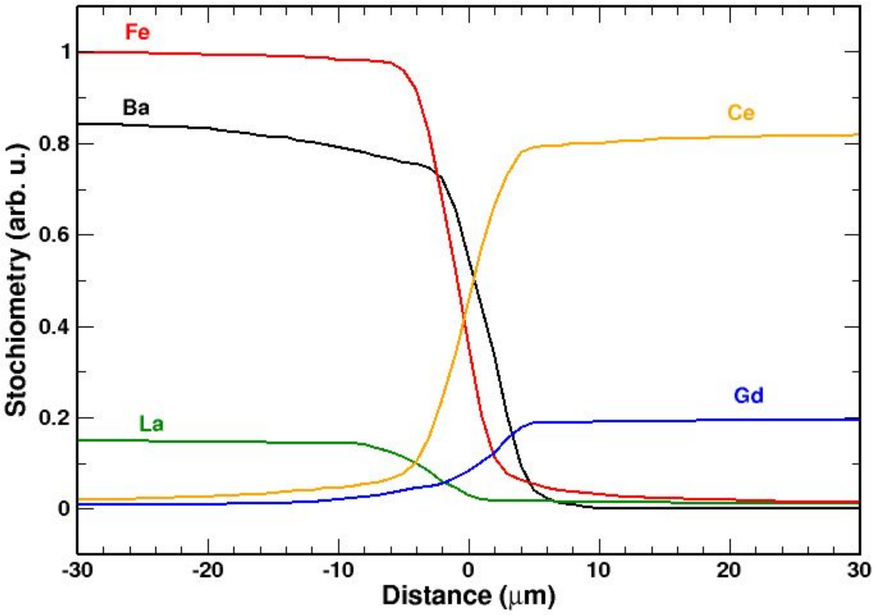

Figure S9 - BL15F/GDC. Concentration profiles renormalized to bulk stoichiometric values.

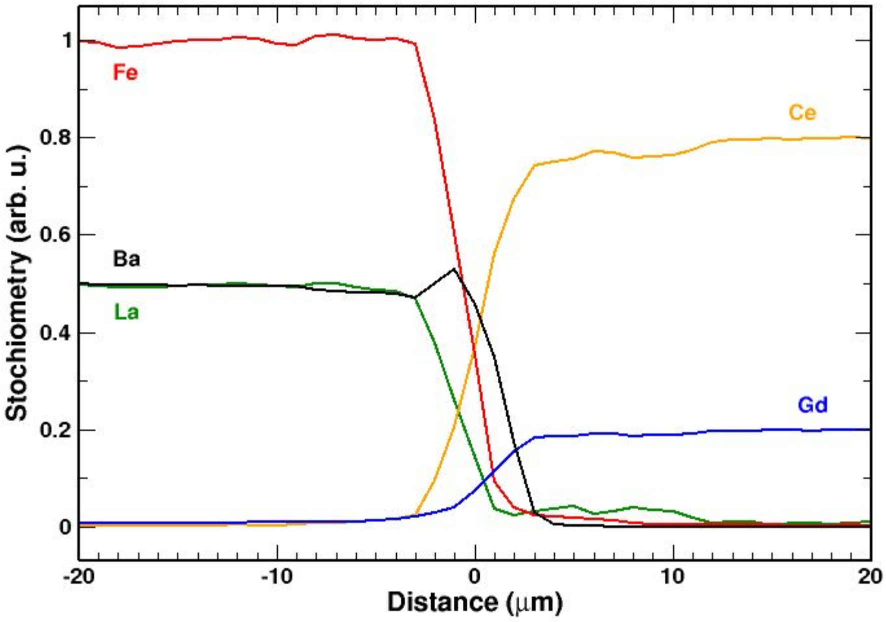

Figure S10 – BL50F/GDC. Concentration profiles renormalized to bulk stoichiometric values.

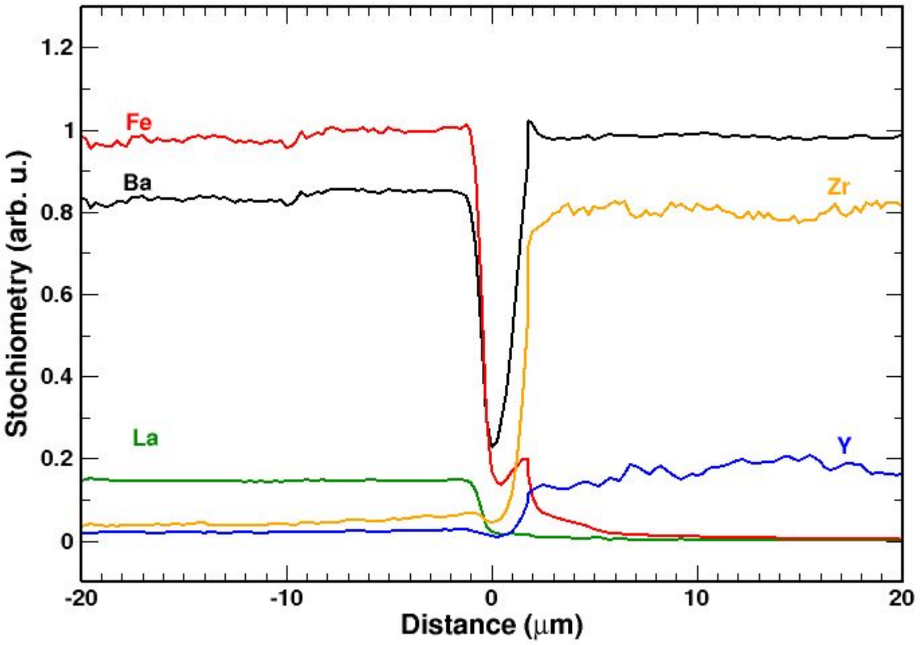

Figure S11 – BL15F/BZY. Concentration profiles renormalized to bulk stoichiometric values.

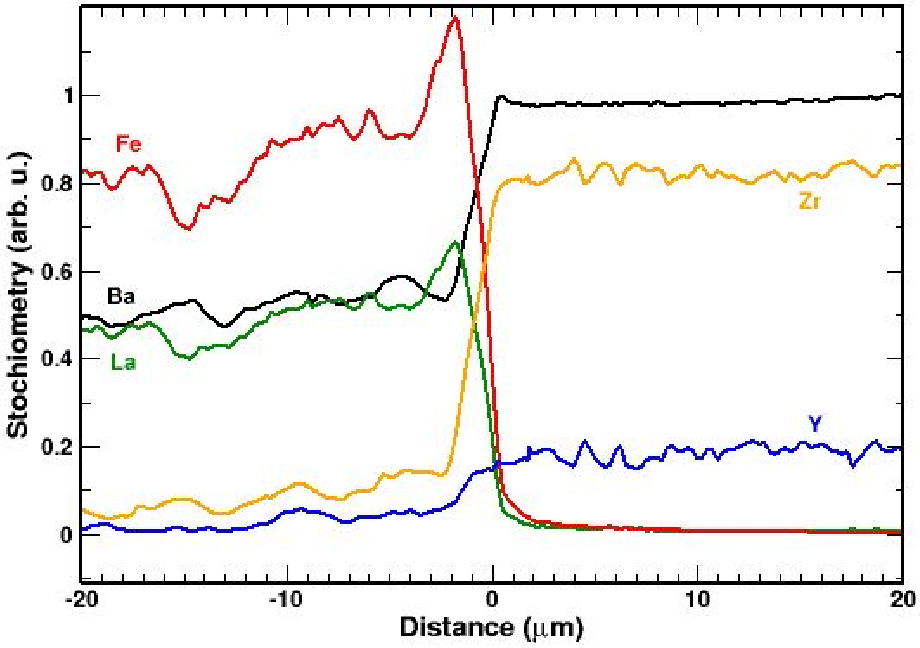

Figure S12 – BL50F/BZY. Concentration profiles renormalized to bulk stoichiometric values.

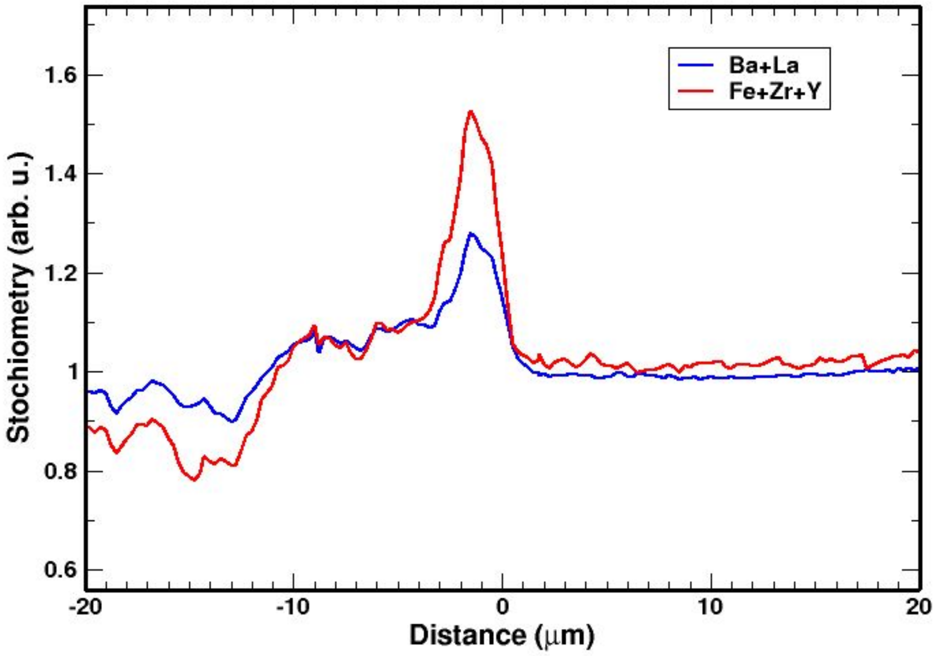

Figure S13 – BL50F/BZY. Sum of concentration profiles renormalized to bulk stoichiometric values for all A-site cations (Ba+La) and for all B-site cations (Fe+Zr+Y). The relative excess of B-site cations (i.e. iron) is evident around (-4,0) μm.

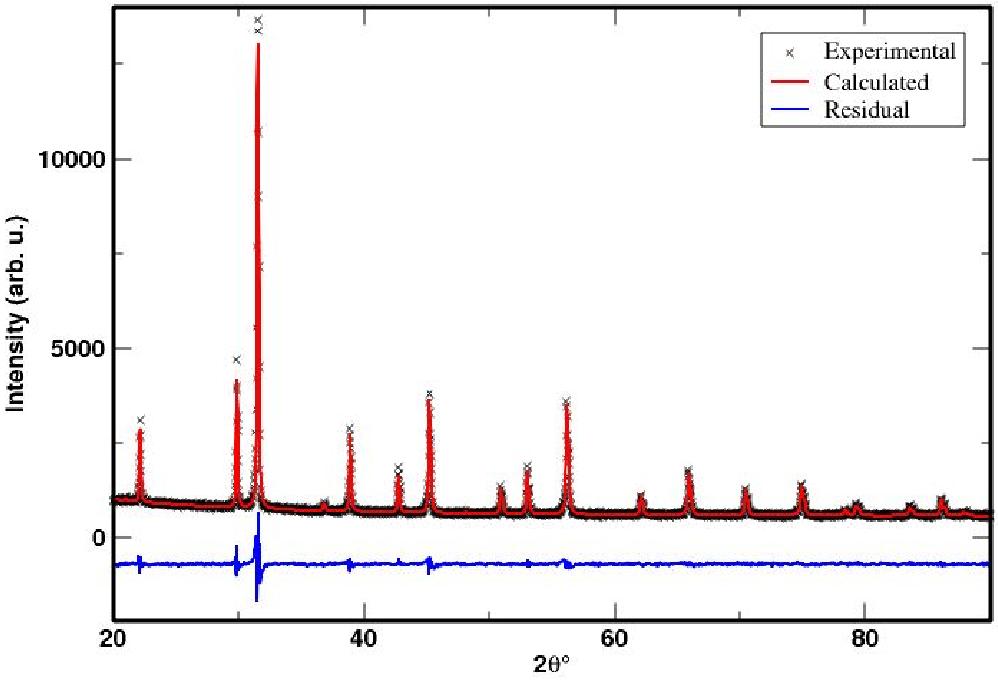

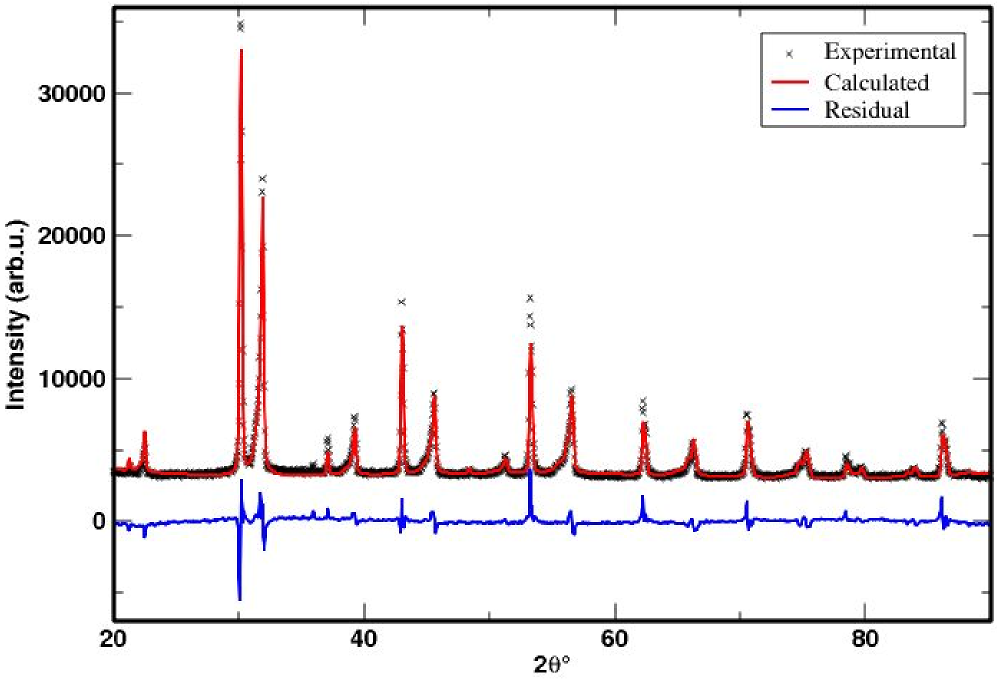

Figure S14 – Rietveld refinement plot for BL5F/BZY (pristine).

Figure S15 – Rietveld refinement plot for BL5F/BZY (12h – 1100°C)

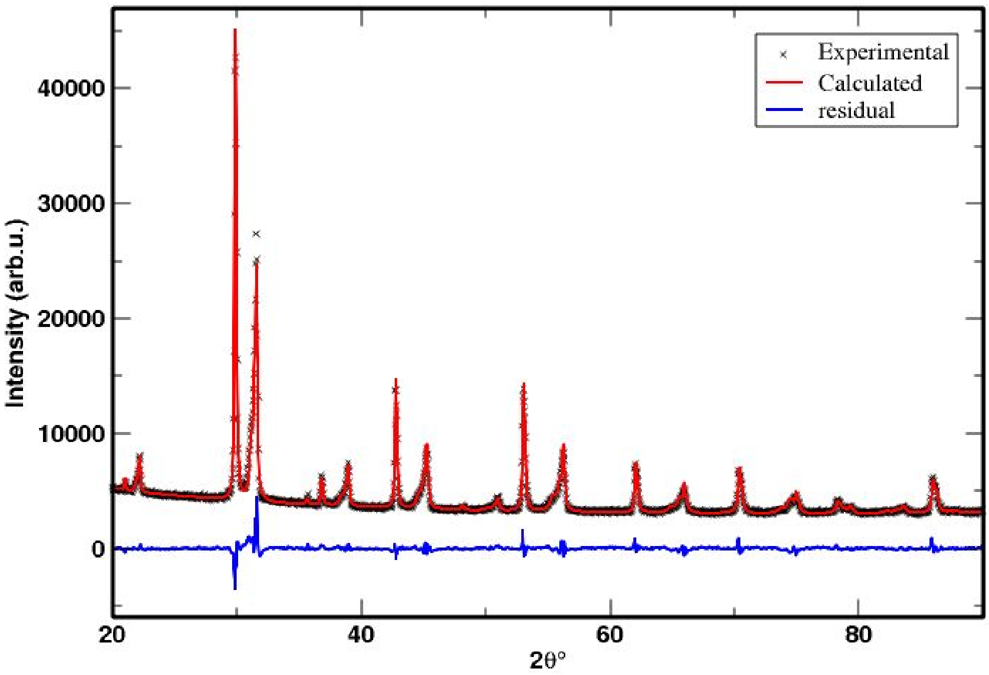

Figure S16 – Rietveld refinement plot for BL5F/BZY (36h – 1100°C)

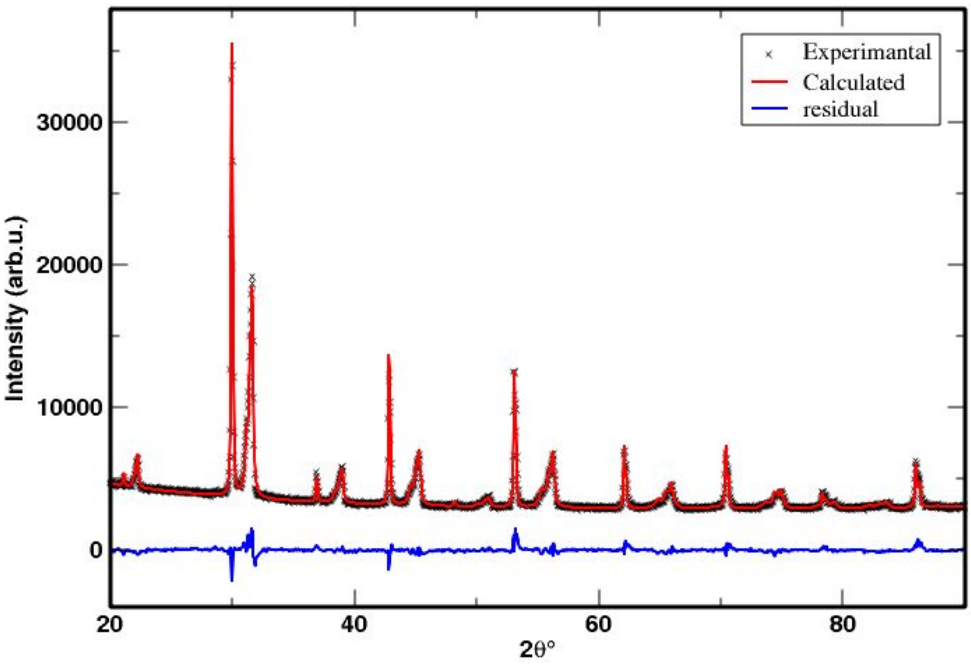

Figure S17 – Rietveld refinement plot for BL5F/BZY (72h – 1100°C)

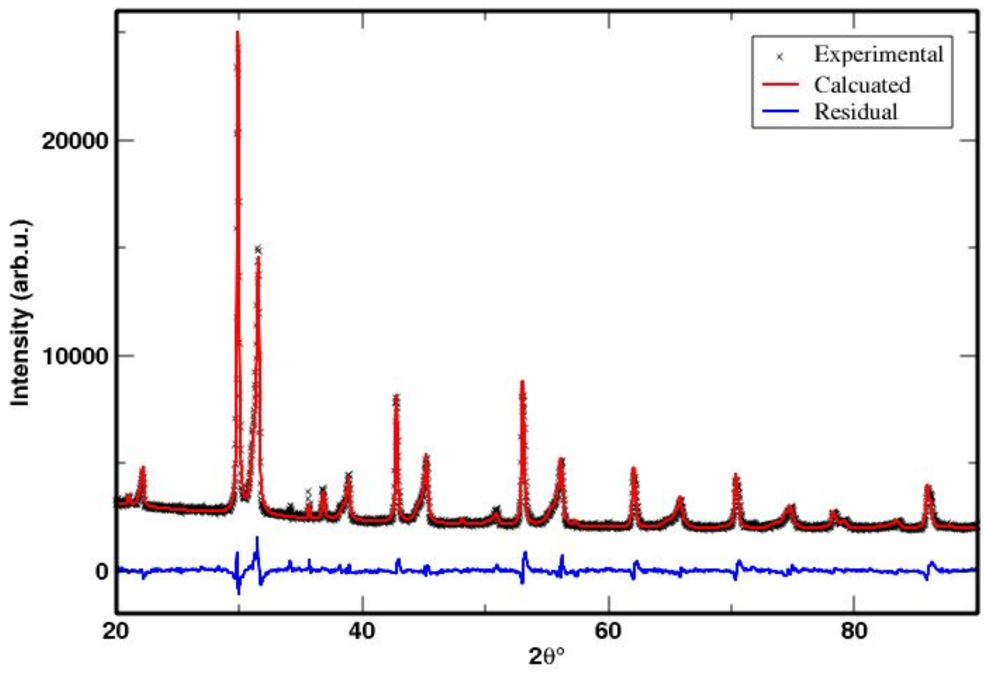

Figure S18 – Rietveld refinement plot for BL5F/BZY (144h – 1100°C)

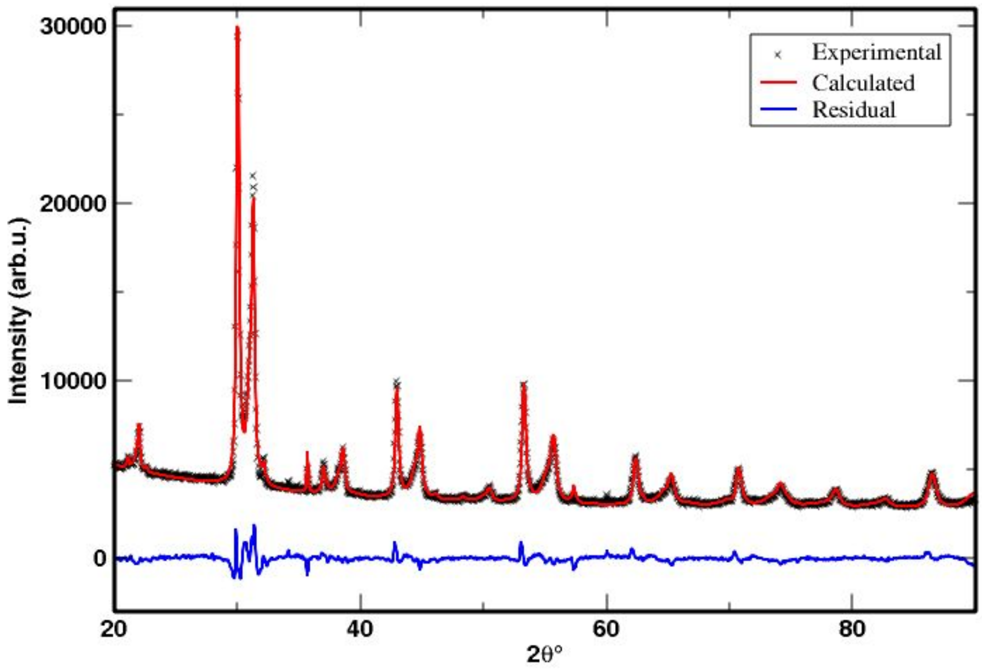

Figure S19 – Rietveld refinement plot for BL5F/BZY (36h – 1300°C)

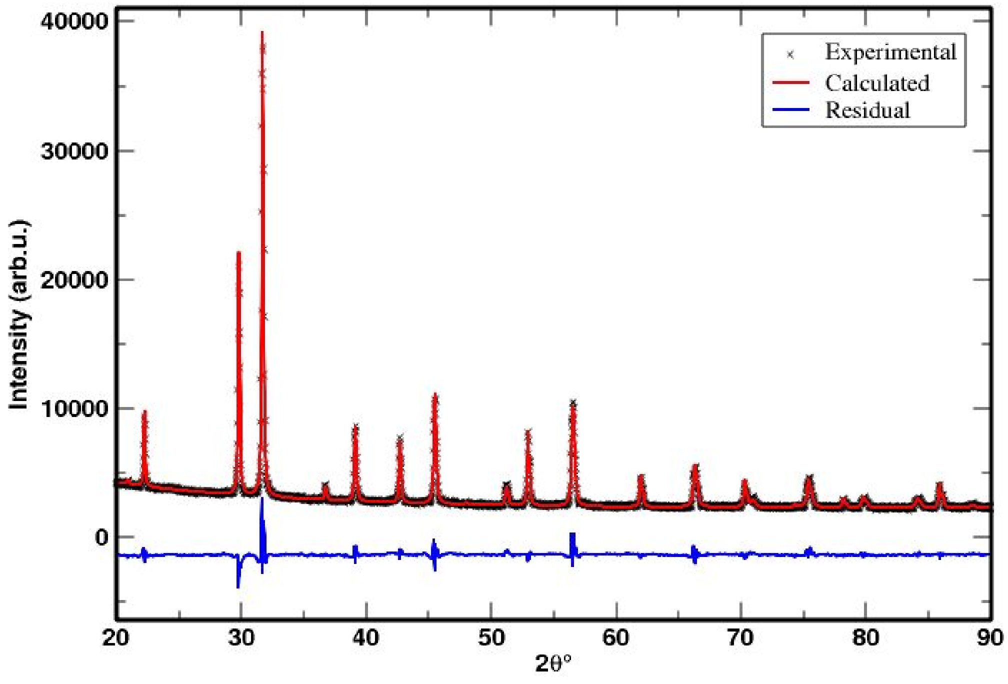

Figure S20 – Rietveld refinement plot for BL15F/BZY (pristine).

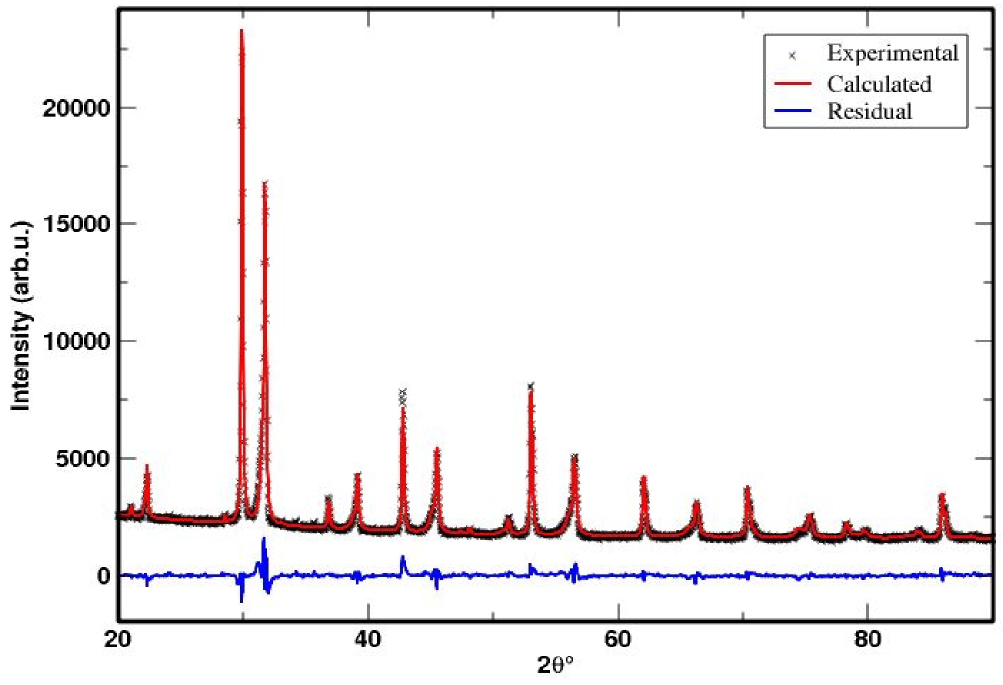

Figure S21 – Rietveld refinement plot for BL15F/BZY (12h – 1100°C)

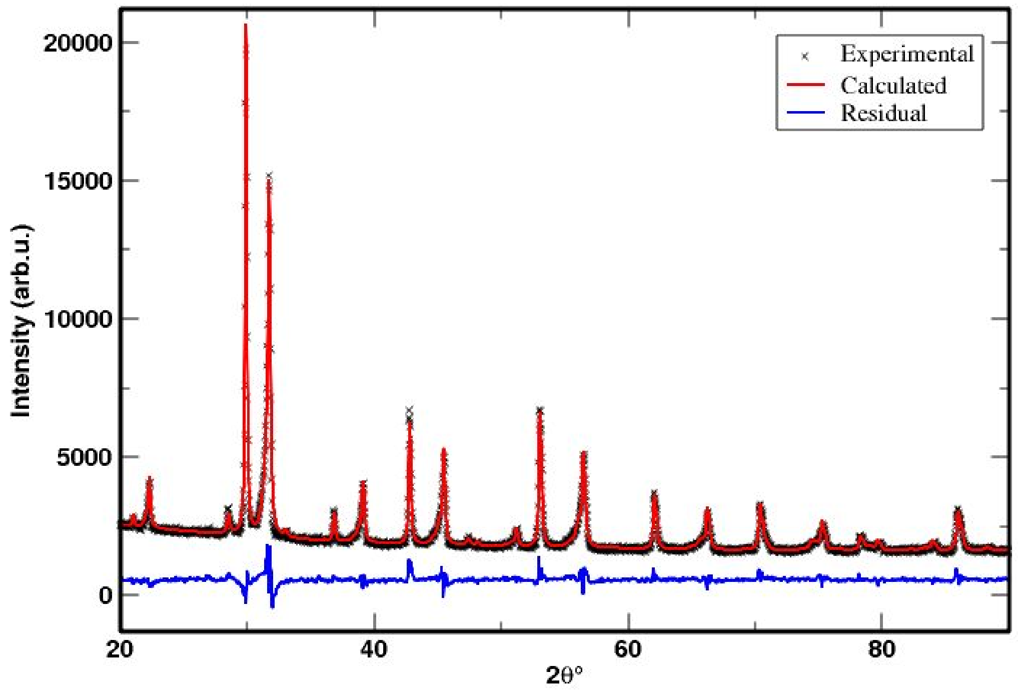

Figure S22 – Rietveld refinement plot for BL15F/BZY (36h – 1100°C)

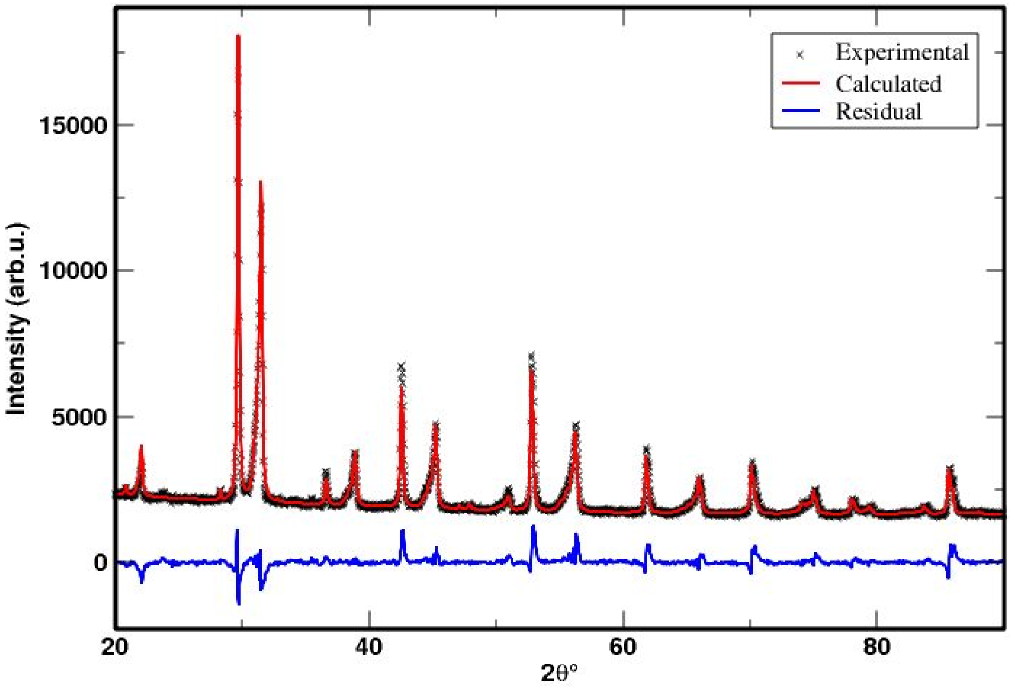

Figure S23 – Rietveld refinement plot for BL15F/BZY (72h – 1100°C)

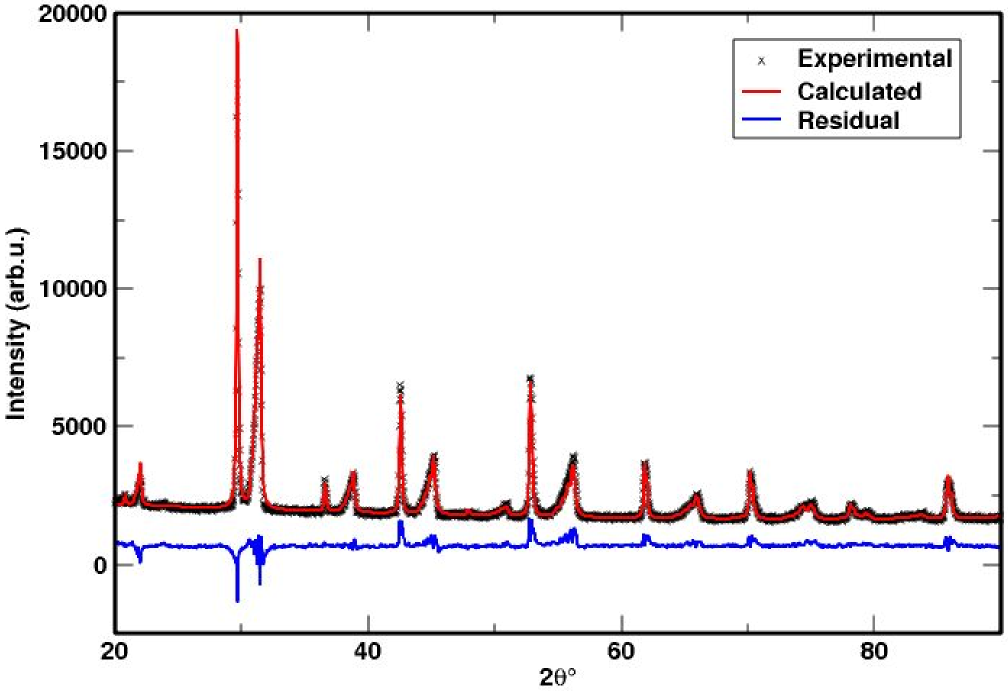

Figure S24 – Rietveld refinement plot for BL15F/BZY (144h – 1100°C)

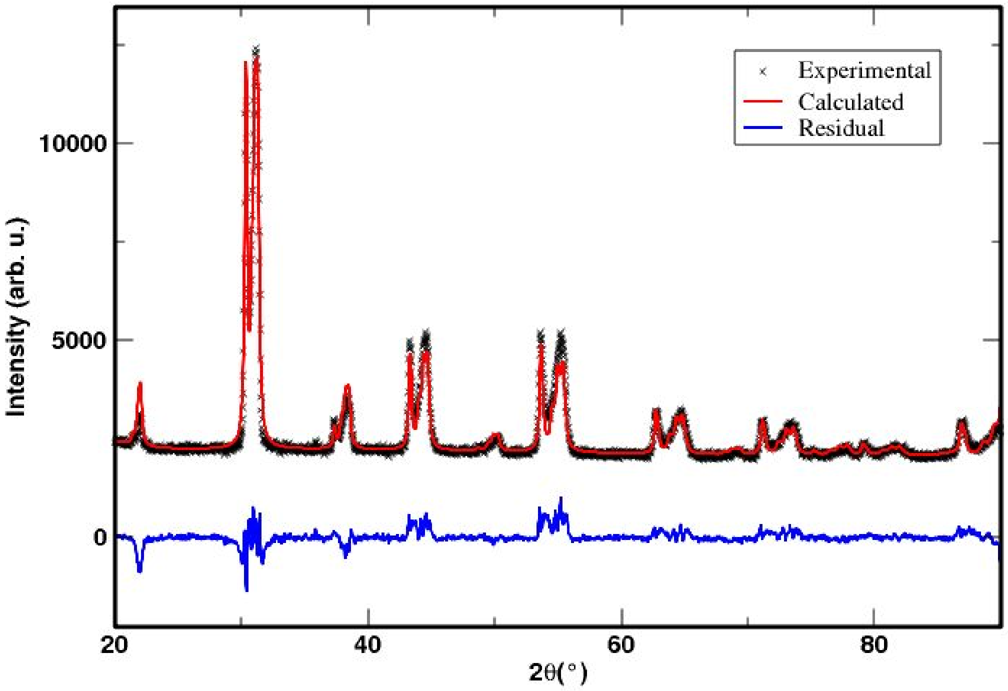

Figure S25 – Rietveld refinement plot for BL15F/BZY (36h – 1300°C)

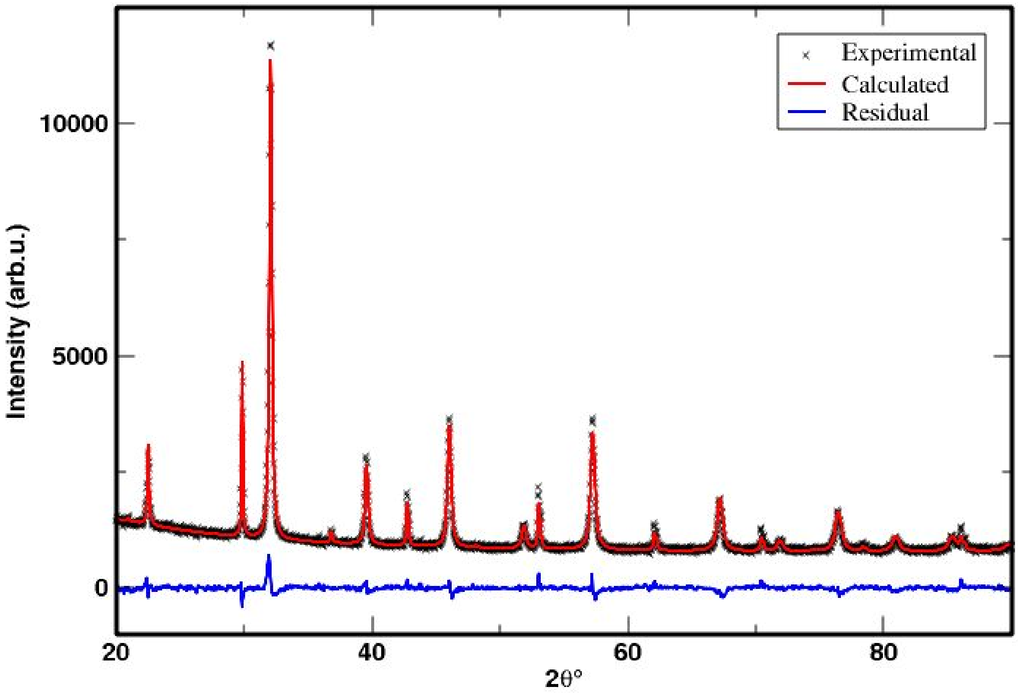

Figure S26 – Rietveld refinement plot for BL50F/BZY (pristine)

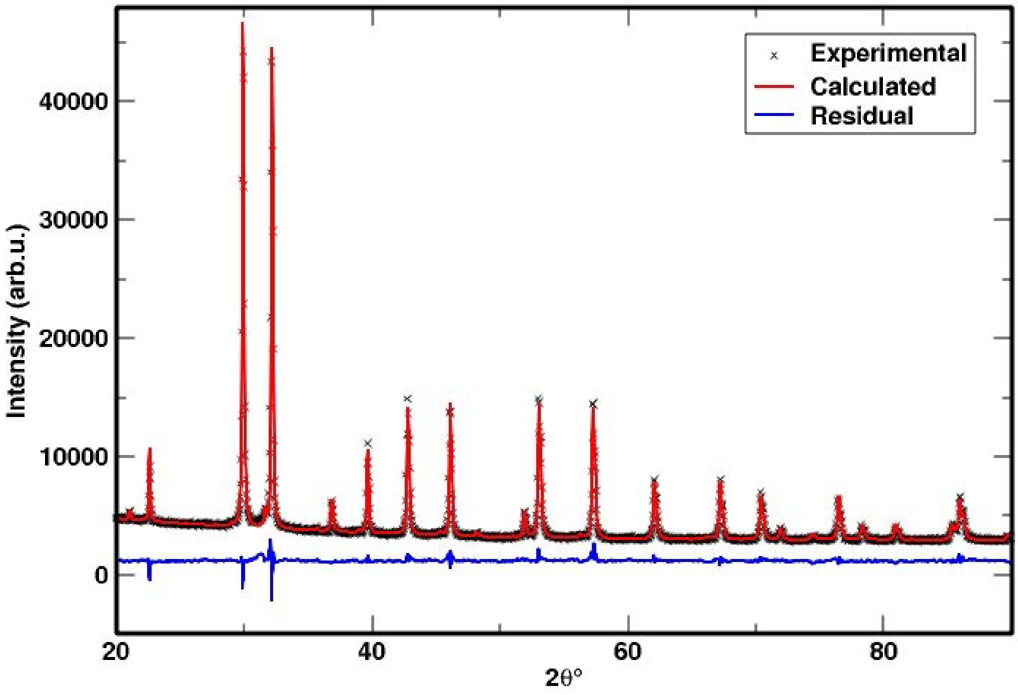

Figure S27 – Rietveld refinement plot for BL50F/BZY (12h – 1100°C)

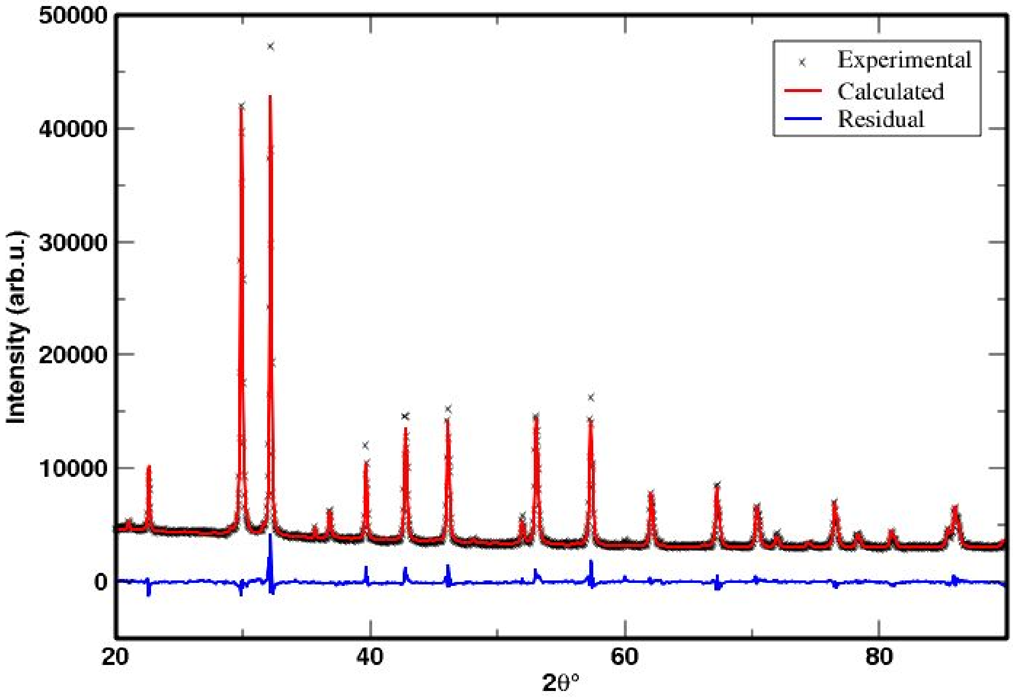

Figure S28 – Rietveld refinement plot for BL50F/BZY (36h – 1100°C)

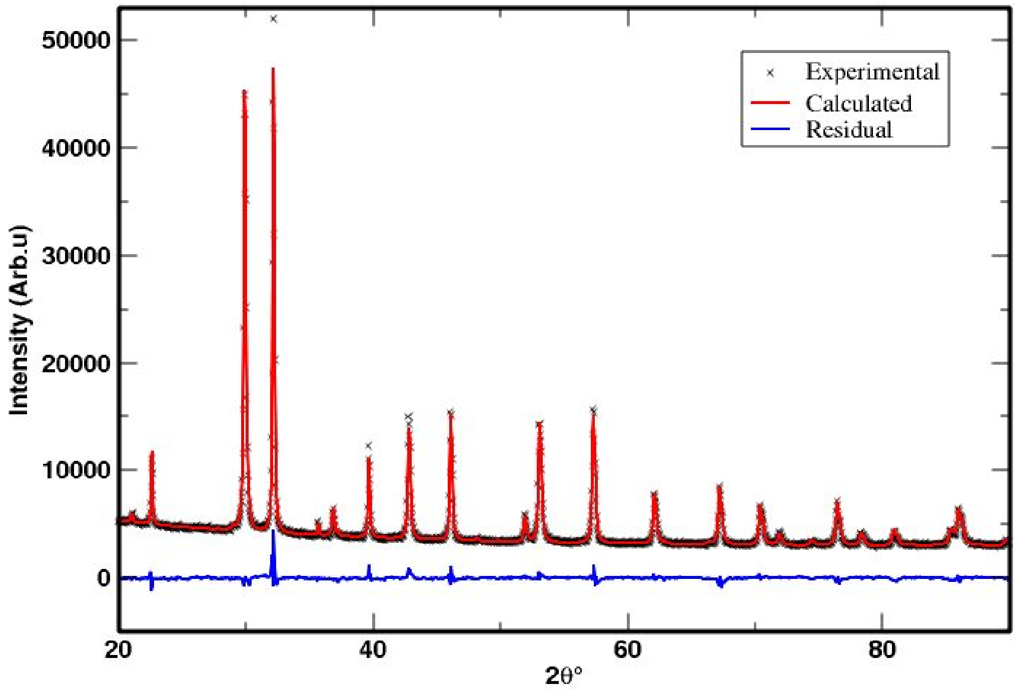

Figure S29 – Rietveld refinement plot for BL50F/BZY (72h – 1100°C)

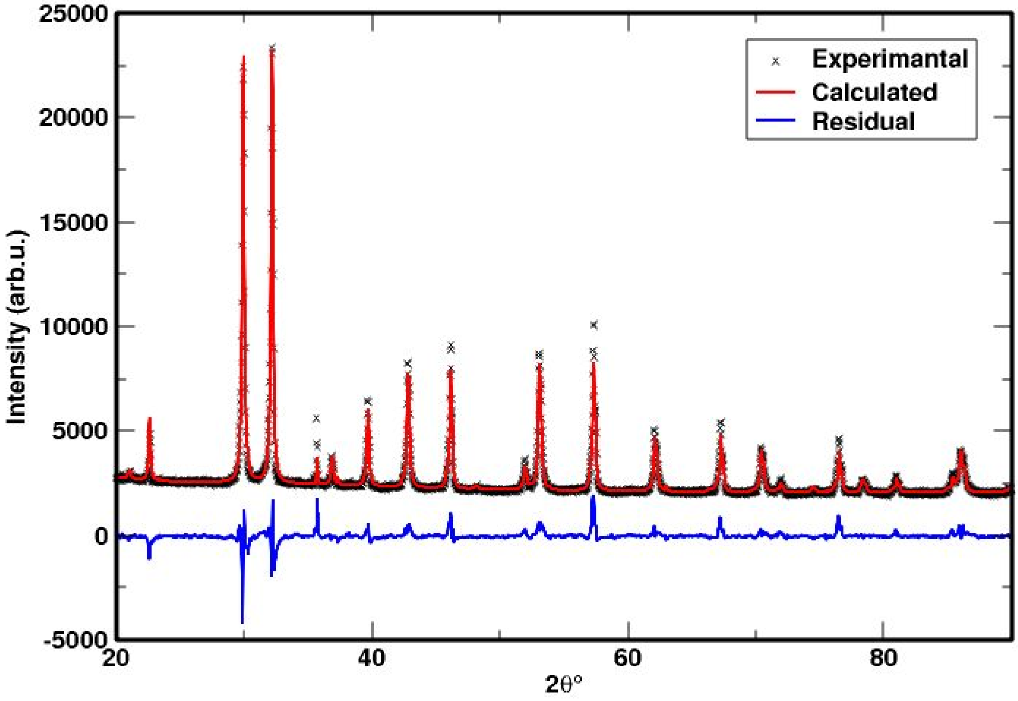

Figure S30 – Rietveld refinement plot for BL50F/BZY (144h – 1100°C)

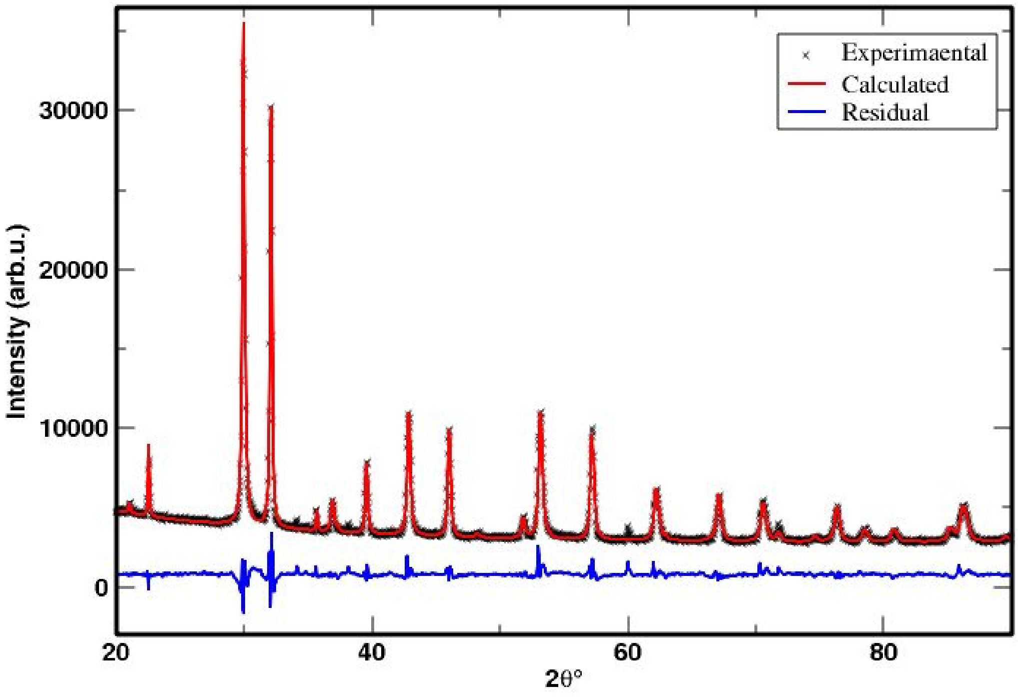

Figure S31 – Rietveld refinement plot for BL50F/BZY (36h – 1300°C)

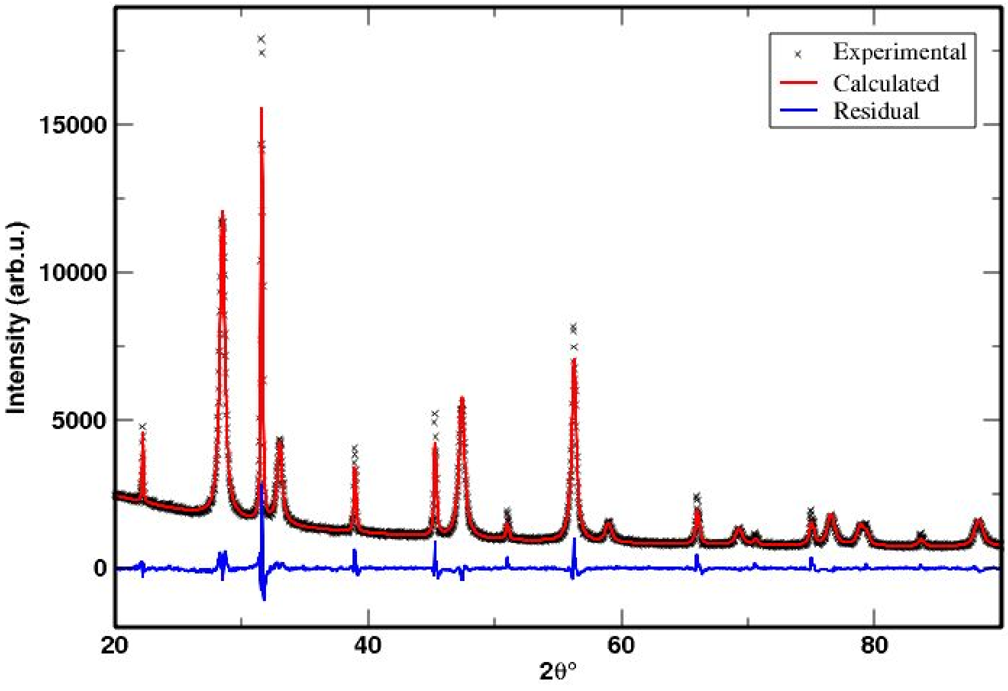

Figure S32 – Rietveld refinement plot for BL5F/GDC (pristine).

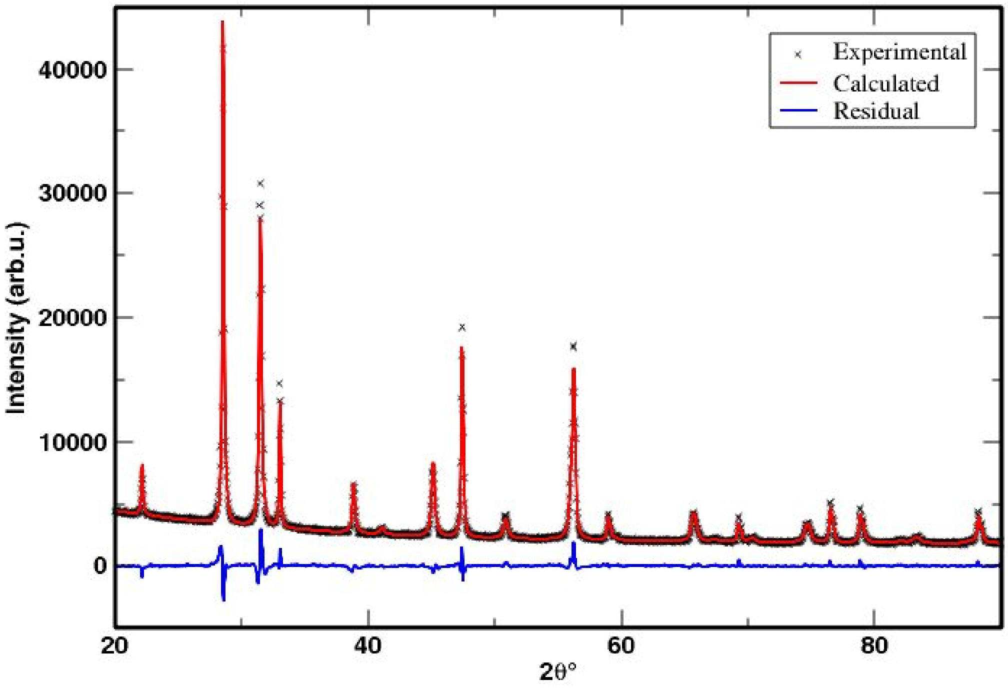

Figure S33 – Rietveld refinement plot for BL5F/GDC (12h – 1100°C)

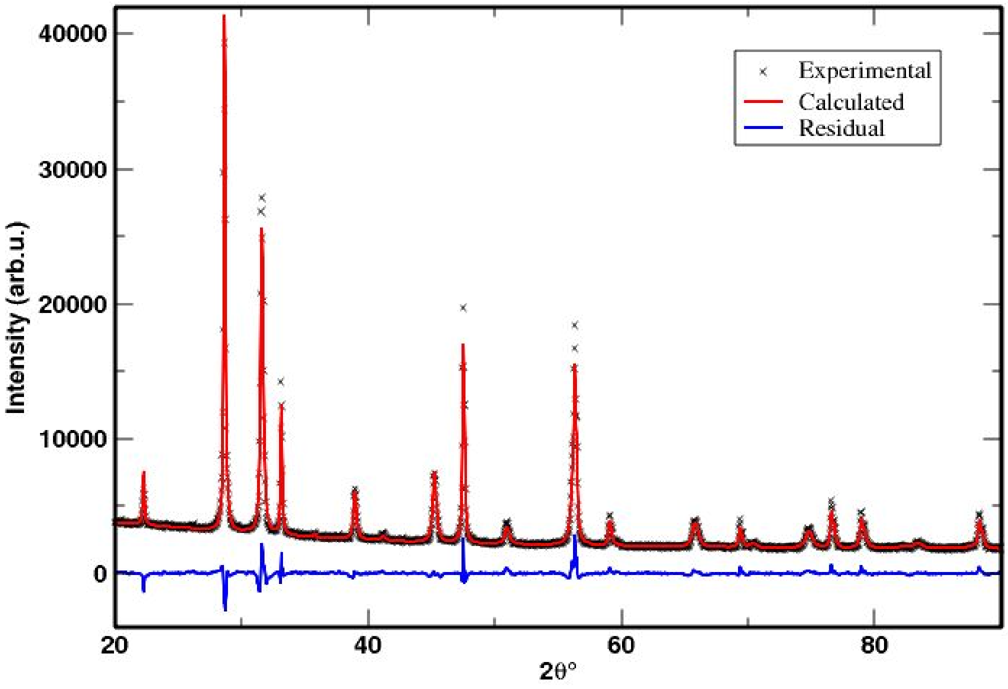

Figure S34 – Rietveld refinement plot for BL5F/GDC (36h – 1100°C)

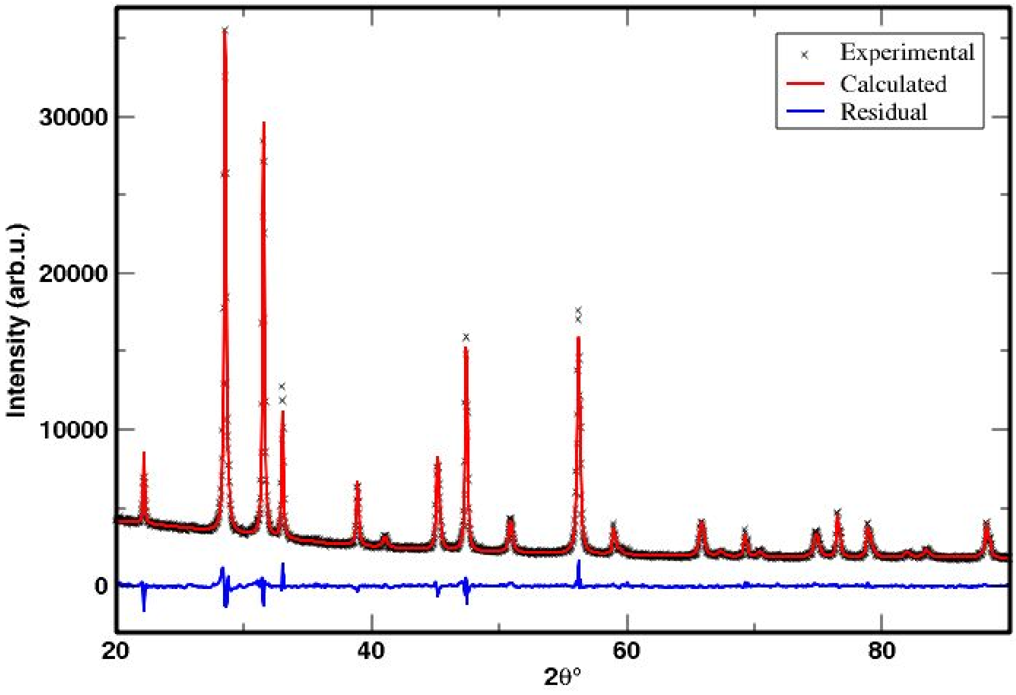

Figure S35 – Rietveld refinement plot for BL5F/GDC (72h – 1100°C)

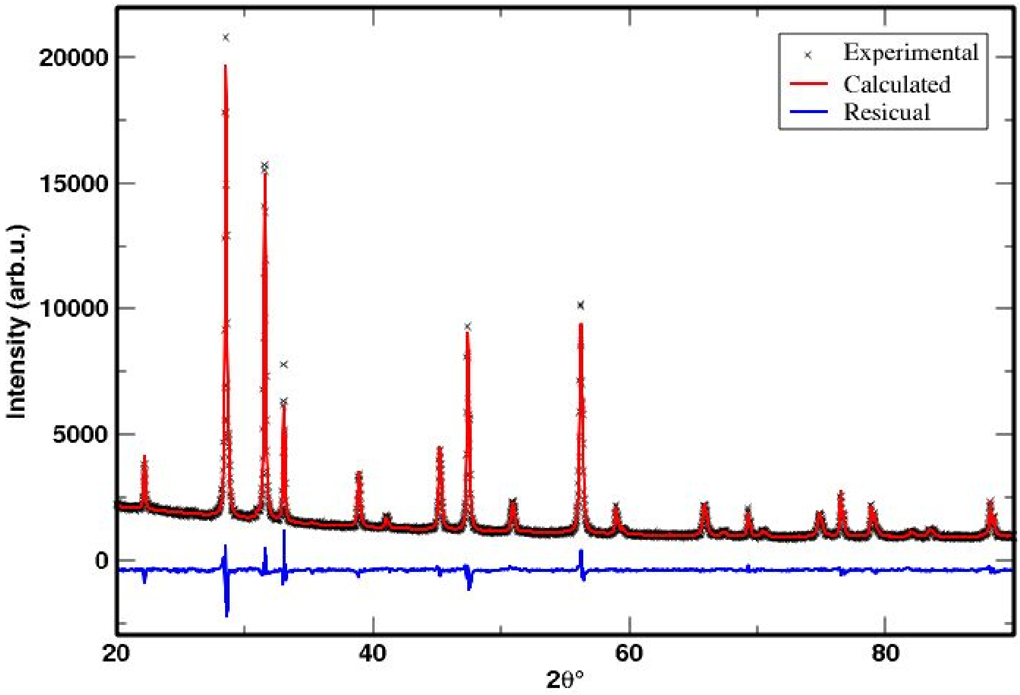

Figure S36 – Rietveld refinement plot for BL5F/GDC (144h – 1100°C)

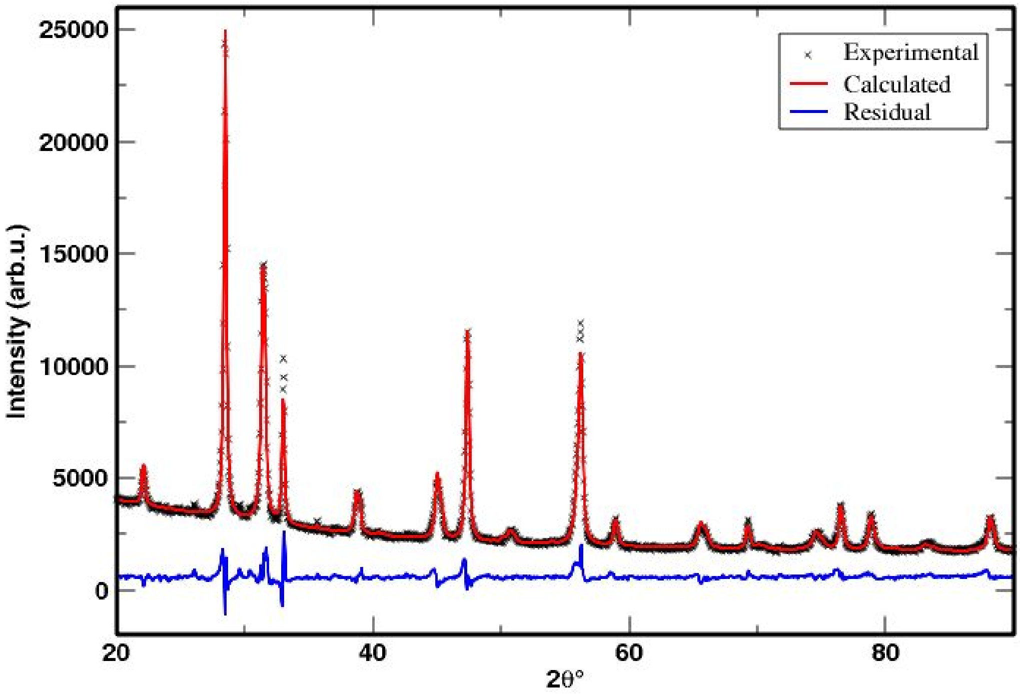

Figure S37 – Rietveld refinement plot for BL5F/GDC (36h – 1300°C)

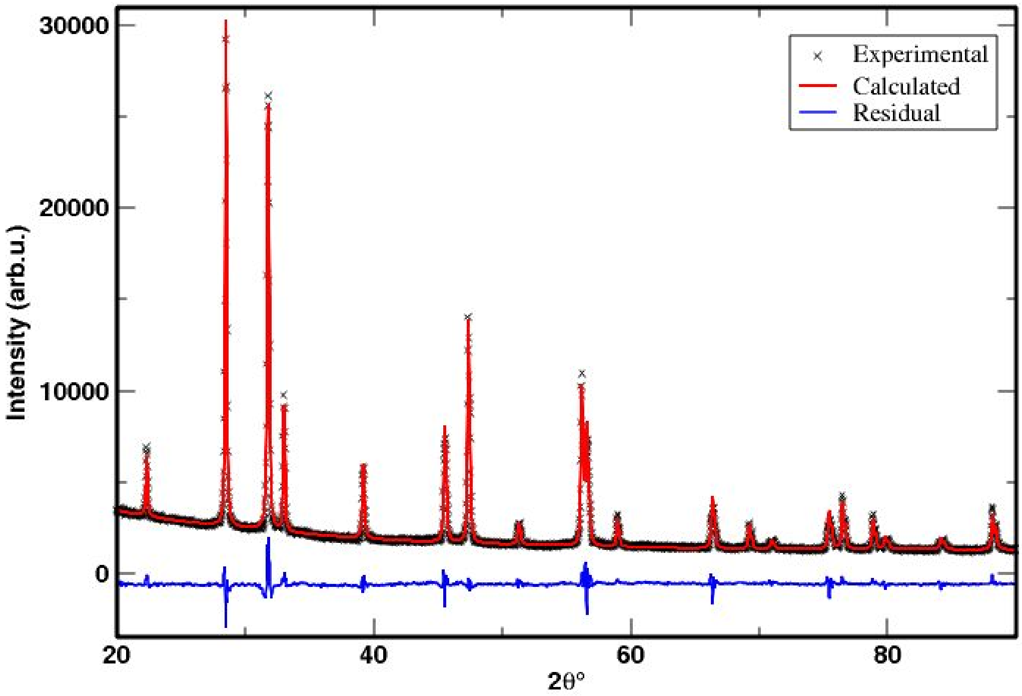

Figure S38 – Rietveld refinement plot for BL15F/GDC (pristine).

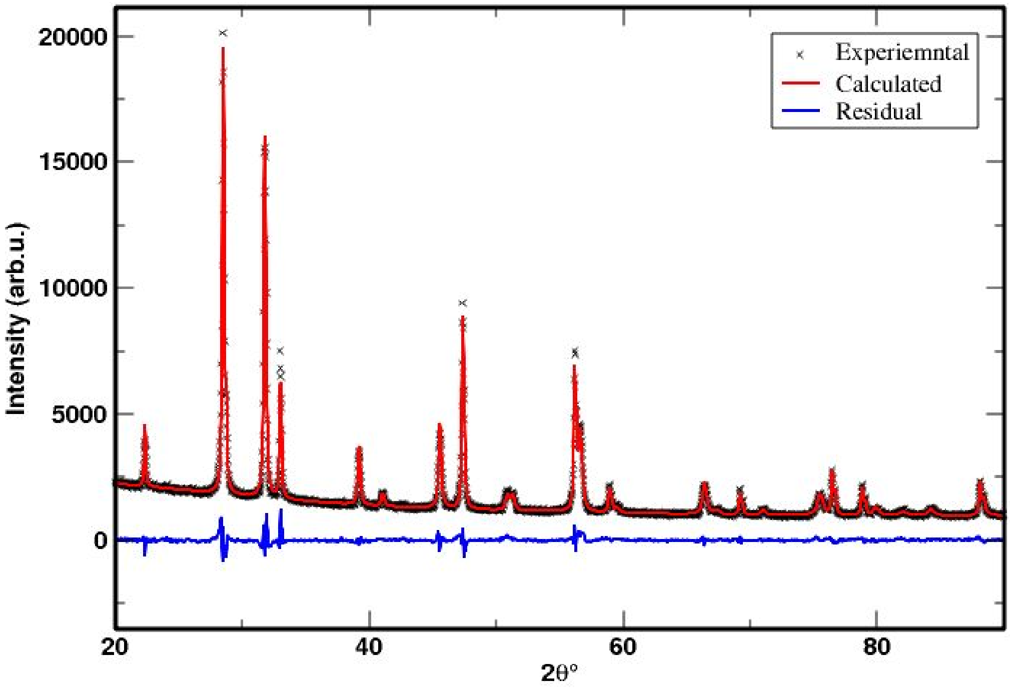

Figure S39 – Rietveld refinement plot for BL15F/GDC (12h – 1100°C)

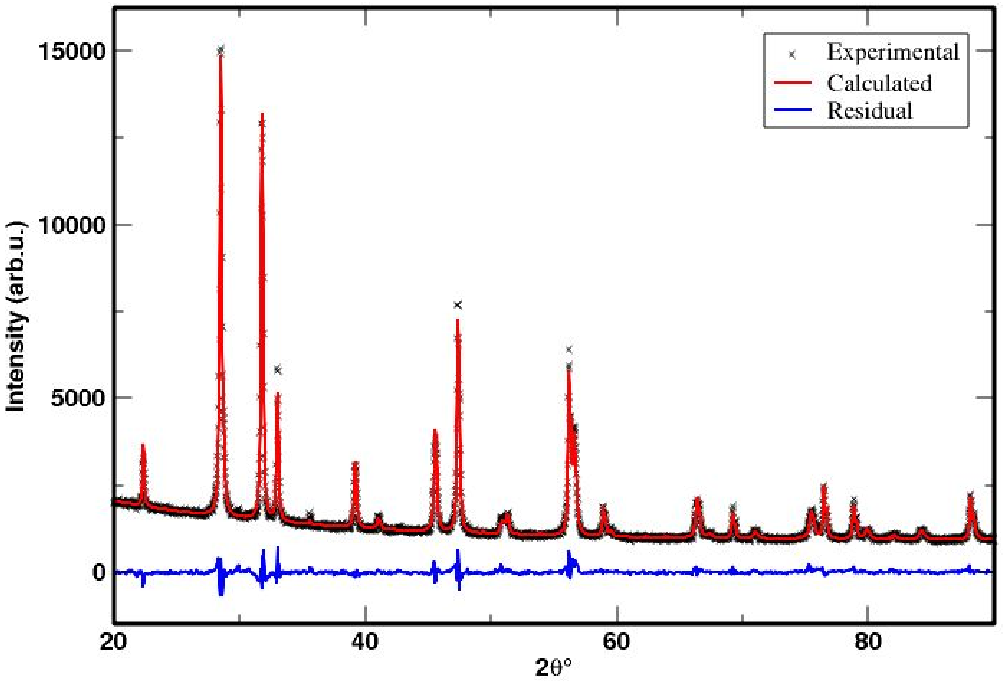

Figure S40 – Rietveld refinement plot for BL15F/GDC (36h – 1100°C)

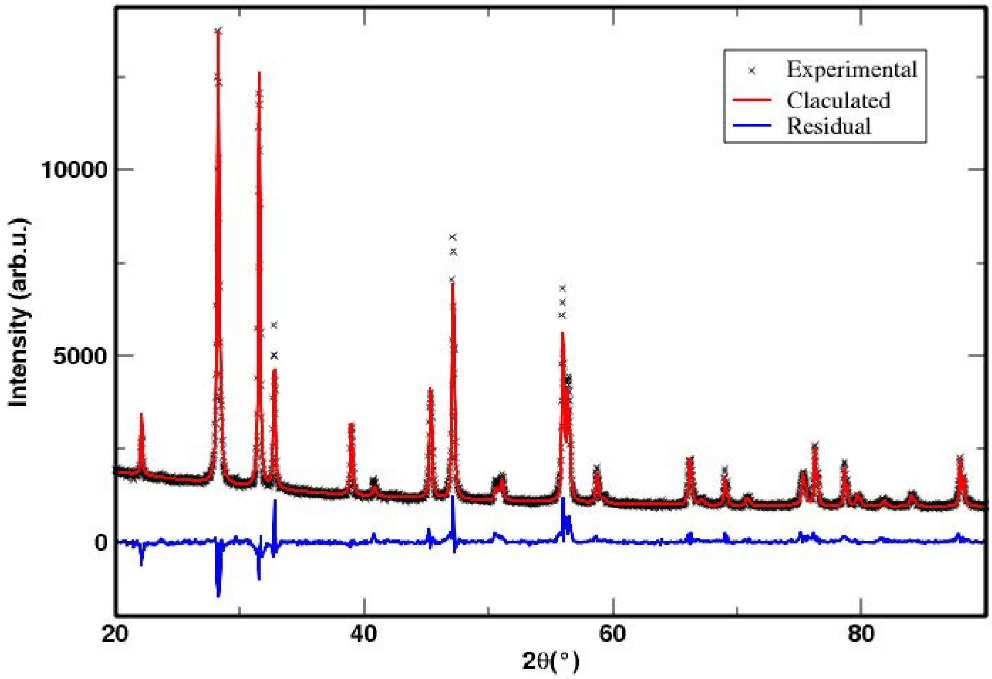

Figure S41 – Rietveld refinement plot for BL15F/GDC (72h – 1100°C)

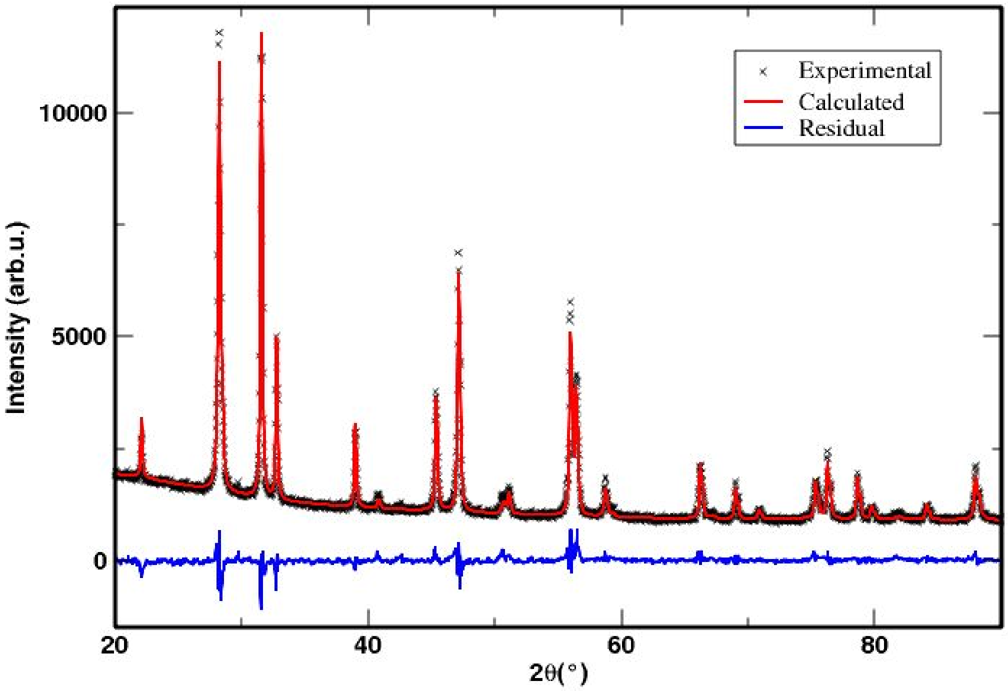

Figure S42 – Rietveld refinement plot for BL15F/GDC (144h – 1100°C)

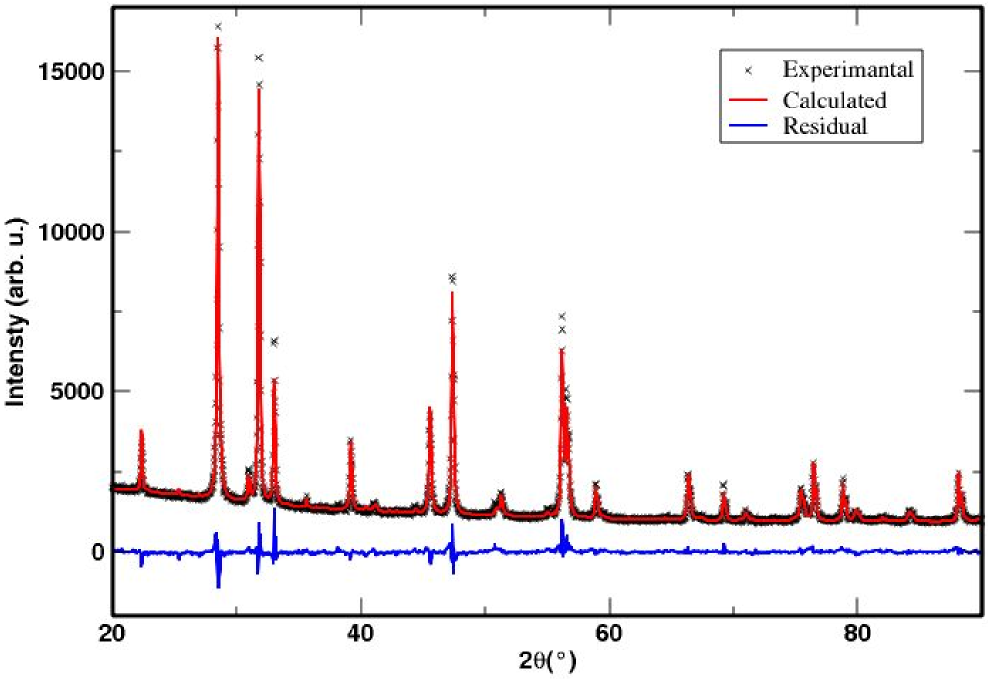

Figure S43 – Rietveld refinement plot for BL15F-GDC (36h – 1300°C)

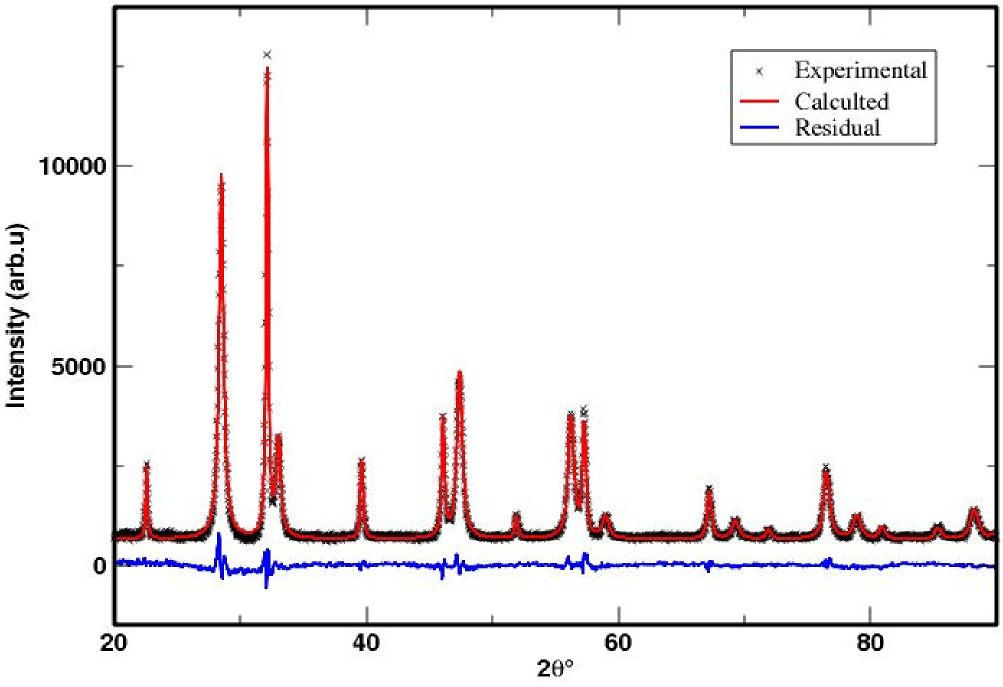

Figure S44 – Rietveld refinement plot for BL50F/GDC (pristine).

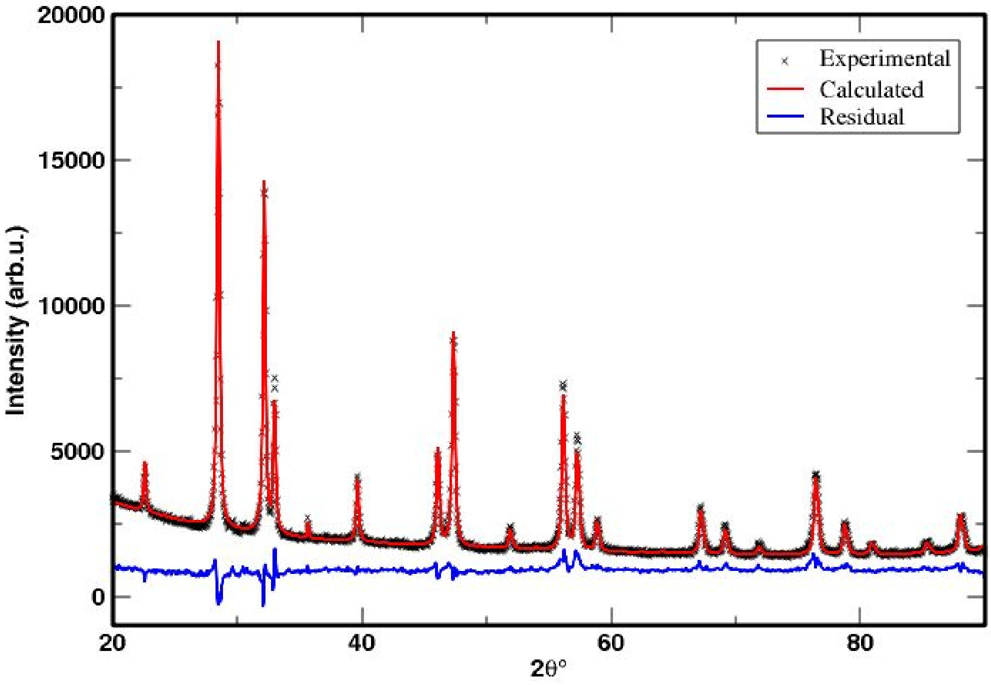

Figure S45 – Rietveld refinement plot for BL50F/GDC (12h – 1100°C)

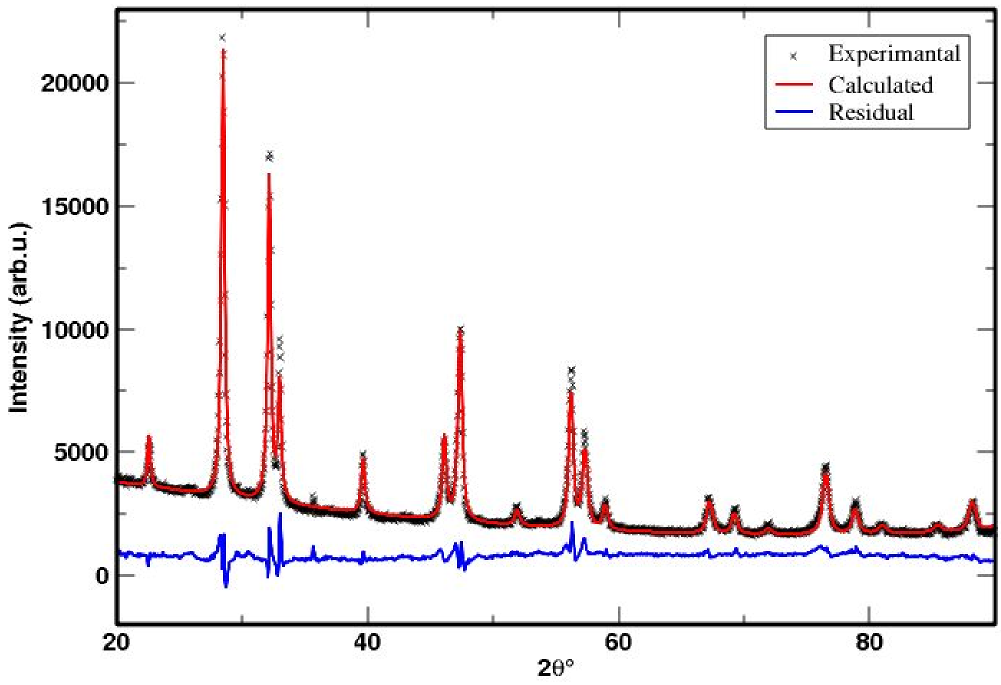

Figure S46 – Rietveld refinement plot for BL50F/GDC (36h – 1100°C)

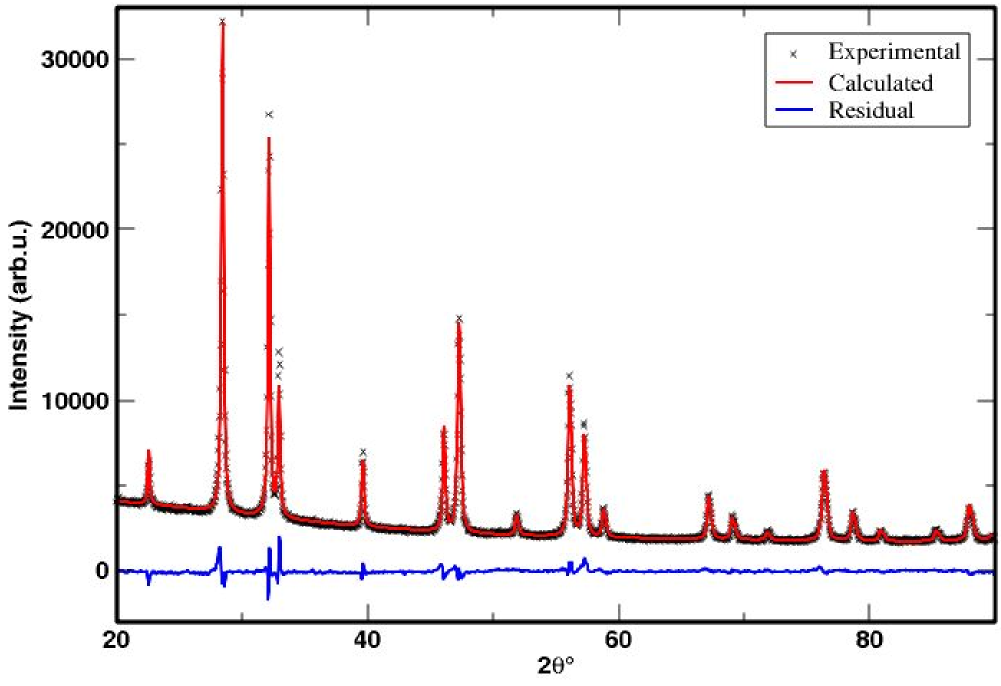

Figure S47 – Rietveld refinement plot for BL50F/GDC (72h – 1100°C)

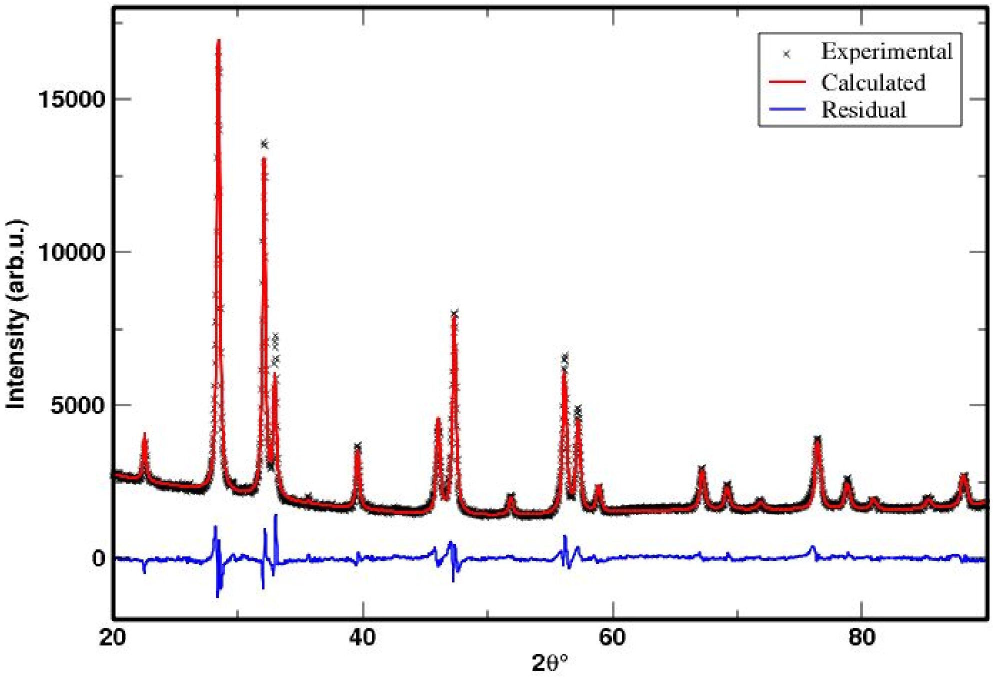

Figure S48 – Rietveld refinement plot for BL50F/GDC (144h – 1100°C)

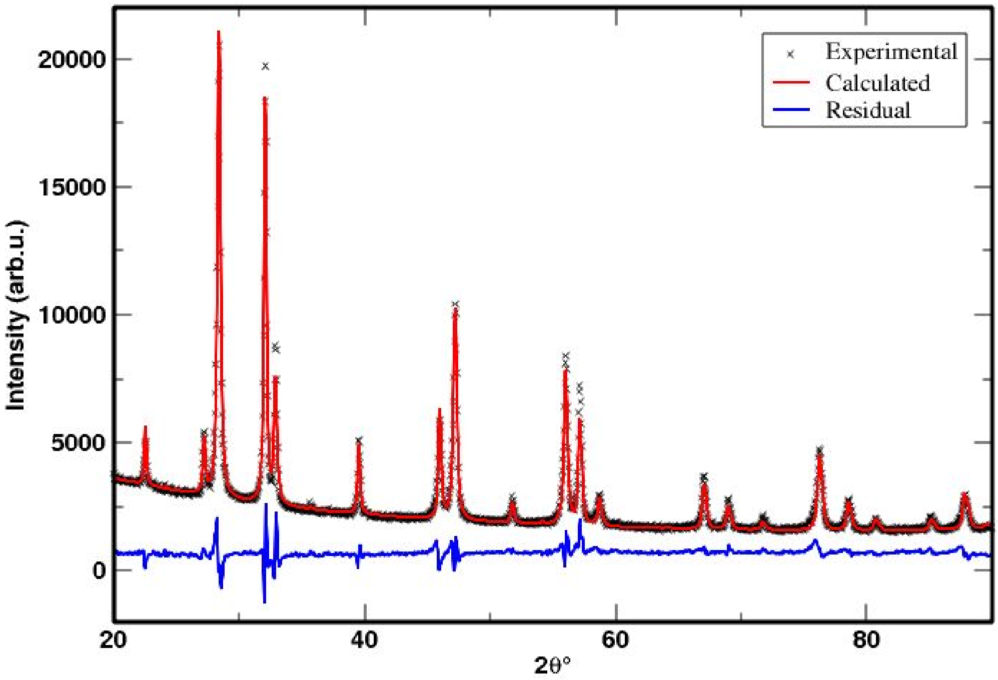

Figure S49 – Rietveld refinement plot for BL50F/GDC (36h – 1300°C)

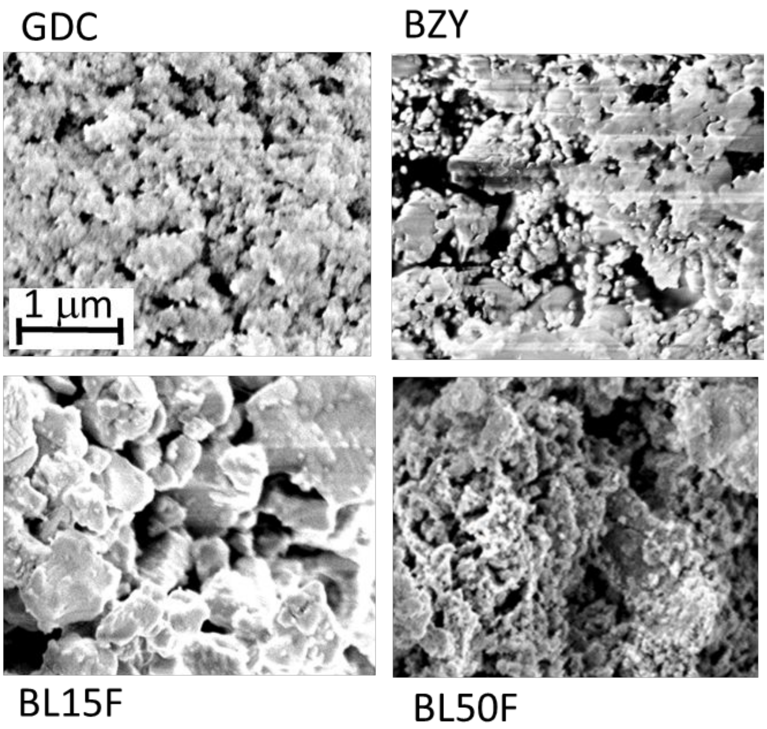

Figure S50 – SEM images of the starting powders. All powders have a sub-micrometer grain size.

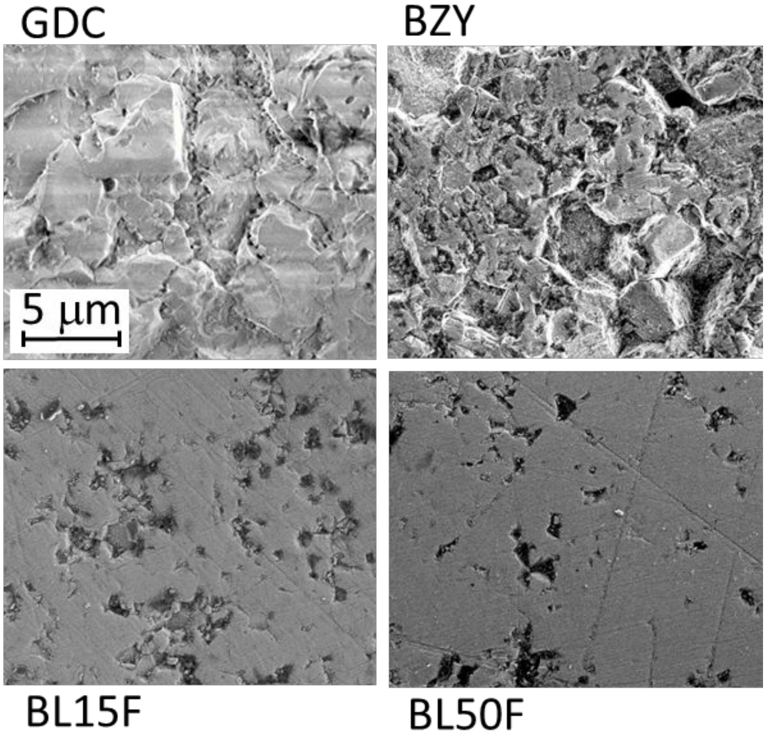

Figure S51 – SEM images of the pellets (GDC, BZY: pellets sintered at 1500 °C (BZY with NiO sintering aid), BL15F, BL50F: BLF zone of the diffusion experiment pellets after 72 h annealing at 1100 °C). GDC is fully dense with ≈ 2-5 μm grain size, BZY has some residual porosity (compare also the intensity fluctuations in Figures 5a and 6b in the main text), with grain size about 1-2 μm. The grains in the BLF pellets are less easily recognizable but also above the micrometer size.
